# Chronic disease burden and health-related quality of life among missouri adults

**DOI:** 10.1007/s11136-026-04422-6

**Published:** 2026-11-01

**Authors:** Sueny Paloma Lima dos Santos, Chester Lee Schmaltz, Jeannette Jackson-Thompson, Eduardo J. Simões

**Affiliations:** 1 Department of Biomedical Informatics, Biostatistics and Medical Epidemiology, Center for Medical Epidemiology and Population Health, School of Medicine, University of Missouri, Columbia, MO USA; 2 MU Institute for Data Science and Informatics, University of Missouri, Columbia, MO USA

**Keywords:** Multimorbidity, Health-related Quality of Life, Chronic disease, SF-36, Population health, Surveys

## Abstract

**Purpose:**

This cross-sectional study examined associations between chronic disease burden, specific chronic conditions, and Short Form 36 (SF-36) health-related quality of life (HRQoL) scores among Missouri adults 18 years of age or older.

**Methods:**

We analyzed survey-weighted data from the 2024 Center for Medical Epidemiology and Population Health Adult Health Survey (*N* = 547). The primary chronic condition count included cardiovascular disease, chronic respiratory disease, arthritis, diabetes, depressive disorder, non-skin cancer, and kidney disease and was categorized as zero, one, or two or more conditions. Obesity was examined as a condition-specific exposure and in sensitivity analyses. Survey-weighted linear regression models adjusted for age, sex, race/ethnicity, educational attainment, employment status, and rurality. The Benjamini–Hochberg procedure was used to control the false discovery rate across the 10 HRQoL outcomes.

**Results:**

Overall, 30.0% of Missouri adults had two or more of the seven selected chronic conditions. Compared with adults with no chronic conditions, adults with two or more conditions had lower scores for nine of the 10 HRQoL outcomes after false discovery rate correction. The largest adjusted differences were observed for Role-Physical (β = −30.65; 95% CI, −46.56 to −14.74), Physical Functioning (β = −28.70; 95% CI, − 38.60 to −18.81), Bodily Pain (β = −28.28; 95% CI, −38.96 to −17.59), Social Functioning (β = −26.18; 95% CI, −35.43 to −16.92), and General Health (β = −24.18; 95% CI, − 30.75 to − 17.62). Physical Component Summary (PCS) was 12.82 points lower (95% CI, −16.98 to −8.66), and Mental Component Summary (MCS) was 6.10 points lower (95% CI, −9.47 to −2.73). Arthritis and cardiovascular disease had the strongest associations with lower PCS, while depressive disorder had the strongest association with lower MCS. In supplementary continuous-count models, each additional condition was associated with a 4.57-point lower PCS score and a 2.20-point lower MCS score.

**Conclusions:**

Greater chronic disease burden was associated with poorer physical and mental HRQoL among Missouri adults. Adding HRQoL assessment to chronic disease surveillance and clinical follow-up may help identify functional limitations and mental health needs that are not apparent from diagnoses alone.

**Supplementary Information:**

The online version contains supplementary material available at https://doi.org/10.1007/s11136-026-04422-6.

## Introduction

Multiple chronic conditions (multimorbidity) are now the norm rather than the exception in primary care [1]. Recent Behavioral Risk Factor Surveillance System (BRFSS) data indicate that 13.1% of Missouri adults reported three or more of eight selected chronic conditions in 2024, compared with 11.3% of U.S. adults [2]. High rates of obesity, heart disease, diabetes, and other conditions contribute to a substantial multimorbidity burden [3]. Prior research has shown that having more than one chronic condition is associated with the poorest level of health-related quality of life (HRQoL) [4, 5]. HRQoL is a multidimensional concept encompassing physical, emotional, and social well-being, and is commonly measured using instruments such as the 36-item Short Form Survey (SF-36) [6].

The SF-36 provides scores in eight domains: Physical Functioning, Bodily Pain, General Health, Vitality, Social Functioning, Mental Health, limitations in work or daily roles due to physical health problems (Role-Physical), and limitations in work or daily roles due to emotional problems (Role-Emotional) [7]. It also yields two summary measures, the Physical Component Summary (PCS) and the Mental Component Summary (MCS). Higher scores in all domains and summary measures indicate better perceived health status. 

Multimorbidity can diminish HRQoL through multiple pathways, including symptom burden, functional limitations, and treatment complexity [8, 9]. However, not all chronic conditions affect quality of life equally [10]. Some conditions (and combinations of conditions) have more pronounced impacts on HRQoL than others [8, 11, 12]. For instance, previous studies have linked arthritis, cardiovascular disease, and chronic respiratory disease with lower physical HRQoL [13, 14]. Mental health conditions, especially depression, are strongly associated with poorer overall health and social well-being. Depression alone has been associated with larger reductions in self-rated health than many chronic physical illnesses, exceeding those reported for angina, arthritis, asthma, or diabetes [15]. Co-occurring mental and physical conditions often exacerbate each other’s effects on quality of life [16].

Although multimorbidity has been widely studied, evidence specific to Missouri remains limited. The state includes a substantial rural population, and differences in demographic, socioeconomic, and healthcare access characteristics may shape both chronic disease burden and HRQoL. We therefore also examined rural–urban differences in multimorbidity and HRQoL among Missouri adults.

We focused on seven noncommunicable condition categories measured through comparable self-reported diagnosis questions and relevant to physical or mental HRQoL: cardiovascular disease, chronic respiratory disease (chronic obstructive pulmonary disease [COPD] or current asthma), arthritis, diabetes, depressive disorder, non-skin cancer, and kidney disease. Using population-weighted survey data from Missouri adults, this study evaluated the associations between overall chronic disease burden and individual conditions and the eight SF-36 domains and the PCS and MCS summary scores. We hypothesized that HRQoL would decline as the number of conditions increased, with depressive disorder showing the strongest associations with mental HRQoL and arthritis or cardiovascular disease showing the strongest associations with physical HRQoL.

## Methods

### Study design and population

We performed a cross-sectional study using data from the 2024 Center for Medical Epidemiology and Population Health (CMEPH) Adult Health Survey (AHS). The CMEPH AHS is a statewide health survey conducted in Missouri to assess adult health status, health behaviors, and chronic conditions among noninstitutionalized adults aged 18 years or older.

The survey used separate landline and cell phone random-digit-dialing (RDD) sampling frames. Telephone numbers were purchased from Marketing Systems Group, the sampling vendor used for the BRFSS and other population health surveys, with a planned allocation of approximately 20% landline and 80% cell phone numbers. For cellular telephone numbers, the eligible adult answering the telephone was selected. For landline households with more than one eligible adult, one adult was selected using the most recent birthday method. Trained interviewers administered the survey using a computer-assisted telephone interviewing (CATI) system after obtaining oral informed consent. The sampling and interviewing procedures followed methods adapted from the Centers for Disease Control and Prevention (CDC) BRFSS data-collection protocol [17]. The survey questionnaire confirms the separate landline and cell phone screening procedures, Missouri residency requirements, and the landline household selection process.

Survey weights were developed using iterative proportional fitting (raking) procedures modeled after CDC BRFSS methodology. Final weights incorporated design and telephone-usage adjustments and were calibrated to external population control totals, including age, sex, race/ethnicity, telephone ownership, and geographic region. The sampling design incorporated geographic and telephone-number density strata. The analytic survey stratum variable combined state, geographic stratum, and density stratum, consistent with the BRFSS structure. The BRFSS documentation defines geographic stratum as identifying the telephone frame and geographic sampling area and density stratum as distinguishing listed and unlisted telephone-number blocks. The final dataset included 547 completed interviews and represented an estimated weighted Missouri adult population of 4,809,135.

The survey response rate was calculated using the American Association for Public Opinion Research Response Rate 4 (AAPOR RR4), consistent with CDC BRFSS methodology. The overall AAPOR RR4 response rate was 21.8%; frame-specific response rates were 25.2% for the landline sample and 20.8% for the cellular telephone sample (Supplementary Table S9).

All analyses incorporated the final raked survey weight (_LLCPWT) and the survey stratum variable to generate population-representative estimates and design-based standard errors. Weighted participant ages ranged from 18 to 93 years, with a weighted mean age of 48.0 years; 51.3% of respondents were female.

The study was approved by the University of Missouri Institutional Review Board (IRB) under the protocol “Population Health Survey” (IRB Project Number 2120187). Initial approval was granted on November 18, 2024. The University of Missouri IRB application served as the study protocol, and the survey questionnaire was included in the approved documentation. The analyses reported in this manuscript were conducted under this approved protocol. The IRB application explicitly identifies itself as the protocol and describes the sampling, recruitment, eligibility, consent, and CATI procedures.

### Measures

The survey collected self-reported histories of chronic health conditions using a series of yes/no questions. Respondents were asked whether a doctor or other health professional had ever told them they had a heart attack; angina or coronary heart disease; stroke; asthma; chronic obstructive pulmonary disease (COPD), including chronic bronchitis or emphysema; arthritis; diabetes; depressive disorder, including depression, major depression, or dysthymia; cancer; or kidney disease, excluding kidney stones or bladder infections. Heart attack, angina or coronary heart disease, and stroke were combined into a cardiovascular disease category. Current asthma and COPD were combined into a chronic respiratory disease category for the primary analyses. In supplementary analyses, current asthma and COPD were also examined separately. Diabetes reported only during pregnancy was excluded. Cancer was defined as non-skin cancer in the primary chronic disease count and condition-specific analyses.

We derived two measures of chronic disease burden: a chronic condition count and specific condition indicators. The primary chronic condition count included seven condition groups: cardiovascular disease, chronic respiratory disease, arthritis, diabetes, depressive disorder, non-skin cancer, and kidney disease. The count ranged from 0 to 7 and was categorized as 0, 1, or 2 or more conditions. This categorization is consistent with common definitions of multimorbidity as the presence of two or more chronic conditions [9]. Obesity was not included in the primary count because it was derived from body mass index calculated from self-reported height and weight, whereas the seven primary conditions were assessed using self-reported diagnosis items. Obesity was defined as body mass index ≥ 30 kg/m^2^. Sensitivity analyses incorporating obesity into the chronic condition count and condition-specific models are described in the Statistical Analysis section.

HRQoL was measured using the Medical Outcomes Study SF-36 Health Survey (version 1). The SF-36 is a validated, widely used questionnaire that assesses eight domains of health status. Survey respondents answered all 36 items, which cover: Physical Functioning (limitations in physical activities such as walking or climbing stairs), Role-Physical (role limitations due to physical health problems, e.g., accomplishing less at work or performing usual daily activities), Bodily Pain (intensity of pain and interference with everyday work), General Health perceptions (overall health and illness outlook), Vitality (energy and fatigue levels), Social Functioning (extent health interferes with social activities), Role-Emotional (role limitations due to emotional problems), and Mental Health (general mental health status, including depression and anxiety). The SF-36 is a validated, widely used questionnaire that assesses eight domains of health status [7]. Each domain score was transformed to a scale ranging from 0 to 100, with higher scores indicating better health status.

We also calculated the PCS and MCS scores using standard norm-based SF-36 scoring algorithms. Domain scores were standardized using U.S. population means and standard deviations, weighted using published factor score coefficients, and transformed into T-scores with a mean of 50 and a standard deviation of 10. Higher PCS and MCS scores indicate better physical and mental health, respectively [7].

Covariates included age in years, sex, race/ethnicity, educational attainment, employment status, and rural or urban residence. Race/ethnicity was categorized as non-Hispanic White, non-Hispanic Black, Hispanic, non-Hispanic Other, or unknown/missing. Educational attainment was grouped as high school or less, some college, or bachelor’s degree or higher. Employment status was grouped as employed or not working. Rural or urban residence was classified using Rural-Urban Commuting Area codes derived from respondents’ ZIP codes and categorized as urban/metro, rural/nonmetro, or unknown. These covariates were selected because they may be associated with both chronic disease burden and HRQoL [18, 19]. The handling of respondents with unknown covariate values is described in the Statistical Analysis section.

For descriptive and age-stratified analyses, age was grouped as 18–44 years, 45–64 years, and 65 years or older. Age was modeled as a continuous variable in the primary regression analyses.

### Statistical analysis

We first described sample characteristics, chronic condition prevalence, and the distribution of chronic disease burden. Categorical variables are presented as unweighted counts and percentages together with survey-weighted percentages. Unweighted percentages were calculated among respondents with nonmissing data for each characteristic. Age is presented as a survey-weighted mean and standard deviation. Weighted means of the eight SF-36 domains and the PCS and MCS scores were calculated by chronic disease burden category to describe unadjusted HRQoL patterns.

The primary chronic disease burden measure included cardiovascular disease, chronic respiratory disease, arthritis, diabetes, depressive disorder, non-skin cancer, and kidney disease. The count was categorized as 0, 1, or 2 or more conditions, with 0 conditions as the reference category. Respondents with unknown values for race/ethnicity, educational attainment, or employment status were excluded from the primary regression analyses because these categories contained only 2, 1, and 3 respondents, respectively. These exclusions represented six unique respondents. Respondents with unknown rurality were retained as a separate category because this group was larger and excluding them could introduce geographic selection bias. The primary analytic sample therefore included 541 respondents before outcome-specific missingness.

We evaluated associations between chronic disease burden and HRQoL using survey-weighted linear regression models fitted separately for each of the eight SF-36 domains, PCS, and MCS. Chronic disease burden was modeled categorically, with zero conditions as the reference group. The primary models included age as a continuous linear term and adjusted for sex, race/ethnicity, educational attainment, employment status, and rurality. Regression coefficients represent adjusted mean differences in SF-36 scores for adults with one condition or two or more conditions compared with adults with no conditions.

We also evaluated associations between individual chronic conditions and HRQoL using the same survey-weighted modeling framework and covariate adjustment. Each condition was entered separately as a binary predictor. Condition-specific exposures included cardiovascular disease, chronic respiratory disease, arthritis, diabetes, depressive disorder, non-skin cancer, kidney disease, and obesity. In supplementary analyses, current asthma and COPD were modeled separately using the same survey-weighted models and covariate adjustment. Regression coefficients represent adjusted mean differences comparing adults with each condition with adults without that condition.

Because 10 HRQoL outcomes were evaluated, we controlled the false discovery rate (FDR) using the Benjamini–Hochberg procedure. For the categorical chronic disease burden models, correction was applied separately across the 10 outcomes for each exposure contrast: one versus zero conditions and two or more versus zero conditions. For the condition-specific models, correction was applied separately across the 10 outcomes within each condition, including the separate current asthma and COPD analyses. Raw p-values and FDR-adjusted q-values are reported.

Several sensitivity analyses were conducted. First, we repeated the primary models using complete cases for all covariates and additionally excluded the 28 respondents with unknown rurality. The complete-case sample included 513 respondents before outcome-specific missingness. Second, to evaluate possible residual confounding from a nonlinear association between age and HRQoL, we repeated the primary models using natural cubic splines for age with three and four degrees of freedom. These models used the same exposure, covariates, survey design, and FDR correction as the primary models. We compared the adjusted two-or-more-versus-zero coefficients from the linear and flexible age specifications. Subpopulation analyses were conducted using survey-aware subsetting of the survey design object, thereby retaining the original survey design information for variance estimation.

Third, because depressive disorder was included in the primary seven-condition count and may conceptually overlap with the SF-36 mental-health outcomes, we reconstructed the chronic condition count after excluding depressive disorder and repeated the survey-weighted models. We also fitted a more stringent specification that included depressive disorder as a separate covariate. In addition, we estimated the adjusted association between depressive disorder and each of the eight SF-36 domains, as well as PCS and MCS.

Fourth, we evaluated the treatment of obesity in two sensitivity analyses. We repeated the chronic disease burden models using an eight-condition count that included obesity. We also repeated the condition-specific models, including obesity as an additional covariate, except when obesity itself was the exposure.

Fifth, we examined the full uncollapsed chronic condition count from 0 to 7 and fitted survey-weighted linear regression models, treating the condition count as a continuous predictor. These models used the same covariate adjustment as the primary analyses. We also fitted a categorical count specification with zero, one, two, three, and four or more conditions, and used design-based Wald tests to evaluate departures from linearity. Benjamini–Hochberg correction was applied across the 10 HRQoL outcomes for the continuous-count models.

For interpretation of effect magnitude, we did not apply a universal 3-to-5-point minimally important difference to all SF-36 outcomes. Because the eight raw SF-36 domains have different variances, adjusted differences were standardized using the survey-weighted standard deviation among adults with no chronic conditions, the reference group in the regression models. Scale-specific standard deviations and half-standard-deviation values were calculated for descriptive interpretation. These standardized values represent cross-sectional differences between groups and were not interpreted as minimally important changes within individuals.

Exploratory analyses described weighted multimorbidity prevalence and weighted means of PCS and MCS by rurality and race/ethnicity. We also tested whether associations between chronic disease burden and PCS or MCS differed by rurality or race/ethnicity using survey-weighted interaction models and design-based Wald tests. These analyses were considered exploratory because the total sample was modest and some race/ethnicity groups contained few respondents. No formal suppression rule was applied; estimates based on sparse subgroup cells were treated as exploratory and interpreted cautiously.

Model assumptions were assessed after model fitting using residual-based diagnostic procedures. The Role-Physical and Role-Emotional domains demonstrated ceiling effects, with a substantial proportion of respondents scoring at the upper limit. These distributional features were considered when interpreting the findings for those domains.

Variance estimation used Taylor series linearization implemented through the R survey package. The survey design incorporated the STRATUM variable and final survey weight (_LLCPWT), and strata containing a single sampling unit were handled using the survey package’s “adjust” option. Results are presented as adjusted mean differences (β) with 95% confidence intervals. Analytic sample sizes varied slightly across outcomes because of outcome-specific missingness. All analyses were conducted using R version 4.5.1 and the survey package. Reporting of the complex sample survey design and analyses was guided by the Preferred Reporting Items for Complex Sample Survey Analysis (PRICSSA) recommendations [20] (Supplementary Table S10).

## Results

### Participant characteristics

A total of 547 adults completed the survey (Table 1). After application of the survey weights, the weighted mean age was 48.0 years (SD, 18.4), and 51.3% of respondents were female. The weighted racial and ethnic distribution was 78.4% non-Hispanic White, 10.5% non-Hispanic Black, 4.1% Hispanic, and 6.6% non-Hispanic Other. Approximately 22.5% of respondents lived in rural or nonmetro areas.

**Table 1:** Unweighted and weighted characteristics of Missouri Adult Health Survey respondents, *N* = 547

Characteristic	Unweighted *n*	Unweighted %	Weighted % (95% CI) or mean (SD)
*Sample characteristics*			
Age, years	547	–	48.0 (18.4)
*Age group*			
18–44 years	184	33.6	45.2
45–64 years	152	27.8	31.7
65 years or older	211	38.6	23.0
*Sex*			
Female	301	55.0	51.3
Male	246	45.0	48.7
*Race and ethnicity*			
Non-Hispanic White	445	81.4	78.4
Non-Hispanic Black	49	9.0	10.5
Hispanic	15	2.7	4.1
Non-Hispanic Other	36	6.6	6.6
Unknown	2	0.4	0.3
*Educational attainment*			
High school or less	175	32.0	32.3
Some college	155	28.3	27.7
Bachelor’s degree or higher	216	39.5	39.8
Unknown	1	0.2	0.2
*Employment status*			
Employed	271	49.5	56.2
Not working	273	49.9	43.7
Unknown	3	0.5	0.1
*Rurality*			
Urban or metro	328	60.0	72.6
Rural or nonmetro	191	34.9	22.5
Unknown	28	5.1	4.8
*Chronic disease burdenᵃ*			
No conditions	184	33.6	45.1
One condition	155	28.3	24.8
Two or more conditions	208	38.0	30.0
*Multimorbidity prevalence within age groupᵇ*			
18–44 years	28	15.2	7.4
45–64 years	55	36.2	41.2
65 years or older	125	59.2	59.1
*Chronic conditions*			
Cardiovascular disease	69	12.6	10.0
Chronic respiratory diseaseᶜ	105	19.2	14.7
Arthritis	218	40.2	32.4
Diabetes	88	16.2	12.3
Depressive disorder	134	24.9	20.4
Non-skin cancer	76	14.0	9.9
Kidney disease	36	6.6	6.6
Obesityᵈ	199	38.0	41.6

Unweighted percentages were calculated among respondents with nonmissing data for each characteristic. Weighted estimates incorporate the final survey weight and stratification. Percentages may not total 100% due to rounding

ᵃ The primary chronic disease count included cardiovascular disease, chronic respiratory disease, arthritis, diabetes, depressive disorder, non-skin cancer, and kidney disease. Obesity was not included in the primary count

ᵇ Multimorbidity was defined as two or more of the seven conditions. In this section, unweighted *n* represents the number of respondents with multimorbidity within each age group, and percentages represent the prevalence of multimorbidity within that age group

ᶜ Chronic respiratory disease was defined as current asthma or chronic obstructive pulmonary disease

ᵈ Obesity was defined as body mass index ≥ 30 kg/m^2^ based on self-reported height and weight. Body mass index was available for 523 respondents. The nonmissing denominators for the other chronic-condition percentages were 547 for cardiovascular and chronic respiratory disease, 542 for arthritis, 543 for diabetes, 539 for depressive disorder, 542 for non-skin cancer, and 543 for kidney disease

Arthritis was reported by 32.4% of the weighted population, followed by depressive disorder (20.4%), chronic respiratory disease (14.7%), diabetes (12.3%), cardiovascular disease (10.0%), non-skin cancer (9.9%), and kidney disease (6.6%). The weighted prevalence of obesity was 41.6%.

Under the primary seven-condition definition, 45.1% of adults had no chronic conditions, 24.8% had one condition, and 30.0% had two or more conditions. When obesity was included as an eighth condition, the weighted prevalence of two or more conditions increased to 41.8% (Supplementary Table S2C, Panel C). The weighted prevalence of multimorbidity under the primary definition increased from 7.4% among adults aged 18–44 years to 41.2% among adults aged 45–64 years and 59.1% among adults aged 65 years or older (Table 1).

### HRQoL by chronic disease burden

Weighted mean SF-36 scores generally declined as chronic disease burden increased (Table 2). Adults with two or more conditions had lower Physical Functioning scores than adults with no conditions (54.6 vs. 91.7), lower Role-Physical scores (44.2 vs. 84.9), lower Bodily Pain scores (51.7 vs. 84.2), and lower General Health scores (50.9 vs. 73.5). Compared with adults with no chronic conditions, adults with two or more conditions had lower weighted mean scores for Vitality (41.7 vs. 61.7), Social Functioning (61.1 vs. 87.1), and Mental Health (70.9 vs. 83.4) (Table 2).

**Table 2:** Weighted health-related quality of life (SF-36) scores by chronic comorbidity burden

SF-36 Domain^a^	0 Conditions	1 Condition	≥ 2 Conditions
Physical functioning	91.7 (15.3); 95% CI 88.7 to 94.8; *n* = 184	81.9 (24.0); 95% CI 74.7 to 89.0; *n* = 155	54.6 (31.2); 95% CI 45.9 to 63.3; *n* = 208
Role-physical	84.9 (30.2); 95% CI 77.2 to 92.6; *n* = 183	76.1 (36.1); 95% CI 66.1 to 86.2; *n* = 154	44.2 (42.5); 95% CI 32.4 to 56.1; *n* = 207
Bodily pain	84.2 (18.4); 95% CI 79.6 to 88.8; *n* = 184	75.9 (21.6); 95% CI 70.2 to 81.6; *n* = 154	51.7 (30.0); 95% CI 42.8 to 60.5; *n* = 208
General health	73.5 (15.4); 95% CI 70.0 to 77.0; *n* = 184	64.3 (21.3); 95% CI 58.1 to 70.5; *n* = 155	50.9 (21.8); 95% CI 45.4 to 56.4; *n* = 208
Vitality	61.7 (21.3); 95% CI 55.3 to 68.1; *n* = 184	53.3 (20.4); 95% CI 47.7 to 58.8; *n* = 155	41.7 (22.8); 95% CI 35.9 to 47.6; *n* = 208
Social functioning	87.1 (17.2); 95% CI 82.9 to 91.4; *n* = 184	85.2 (21.9); 95% CI 80.6 to 89.8; *n* = 155	61.1 (31.5); 95% CI 52.4 to 69.8; *n* = 208
Role-emotional	78.5 (30.1); 95% CI 70.7 to 86.2; *n* = 183	82.7 (31.0); 95% CI 76.1 to 89.4; *n* = 154	66.9 (36.8); 95% CI 57.1 to 76.6; *n* = 207
Mental health	83.4 (15.1); 95% CI 79.4 to 87.4; *n* = 184	79.3 (16.4); 95% CI 75.7 to 83.0; *n* = 155	70.9 (20.8); 95% CI 66.1 to 75.8; *n* = 208
Physical component summary	52.3 (7.5); 95% CI 50.4 to 54.1; *n* = 182	47.4 (10.2); 95% CI 44.5 to 50.3; *n* = 153	36.0 (13.3); 95% CI 32.1 to 39.9; *n* = 206
Mental component summary	51.3 (8.9); 95% CI 48.8 to 53.8; *n* = 182	51.2 (10.5); 95% CI 48.9 to 53.5; *n* = 153	47.8 (11.0); 95% CI 45.1 to 50.5; *n* = 206

^a^SF 36 domain scores range from 0, indicating worst health, to 100, indicating best health. PCS and MCS are norm-based scores with a mean of 50 and an SD of 10. Values are survey-weighted mean (weighted SD), 95% confidence interval, and unweighted n. Confidence intervals were estimated using the stratified survey design and final survey weight

The mean PCS score decreased from 52.3 among adults with no conditions to 47.4 among adults with one condition and 36.0 among adults with two or more conditions. The mean MCS score was similar among adults with no conditions and adults with one condition, at 51.3 and 51.2, respectively, but was lower among adults with two or more conditions, at 47.8 (Table 2).

**Fig. 1:**
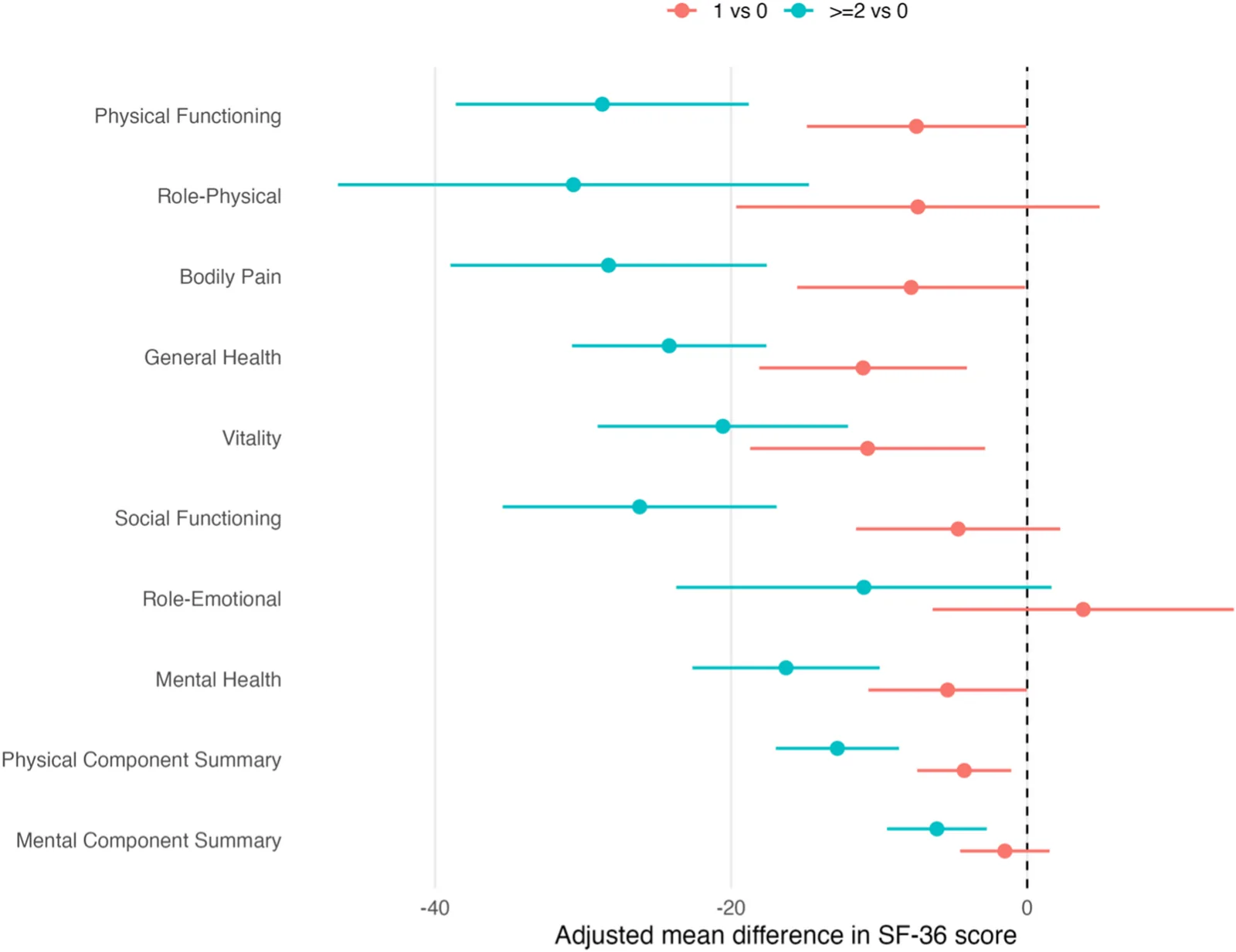
Adjusted mean differences in SF-36 scores by chronic disease burden. Points represent adjusted β coefficients from survey-weighted linear regression models comparing adults with one chronic condition or two or more chronic conditions to those with no chronic conditions (reference group). Horizontal lines indicate 95% confidence intervals. Models were adjusted for age, sex, race/ethnicity, education, employment status, and rural/urban residence. Negative values indicate lower health-related quality of life

After excluding six respondents with sparse unknown values for race/ethnicity, educational attainment, or employment status, the primary regression sample included 541 respondents before outcome-specific missingness. Respondents with unknown rurality were retained as a separate category. After adjustment for age, sex, race/ethnicity, educational attainment, employment status, and rurality, greater chronic disease burden remained associated with poorer HRQoL (Table 3; Fig. 1).

**Table 3:** Survey-weighted adjusted associations between chronic disease burden and SF-36 scores

Domain	Analytic *n*	Comparison	Adjusted β	95% CI	*p*	q (FDR)
Physical functioning	541	1 vs. 0	−7.48	−14.89, −0.07	0.048	0.081
		≥ 2 vs. 0	−28.70	−38.60, −18.81	< 0.001	< 0.001
Role-physical	538	1 vs. 0	−7.38	−19.66, 4.90	0.238	0.298
		≥ 2 vs. 0	−30.65	−46.56, −14.74	< 0.001	< 0.001
Bodily pain	540	1 vs. 0	−7.84	−15.54, −0.14	0.046	0.081
		≥ 2 vs. 0	−28.28	−38.96, −17.59	< 0.001	< 0.001
General health	541	1 vs. 0	−11.09	−18.10, −4.07	0.002	0.020
		≥ 2 vs. 0	−24.18	−30.75, −17.62	< 0.001	< 0.001
Vitality	541	1 vs. 0	−10.78	−18.71, −2.84	0.008	0.029
		≥ 2 vs. 0	−20.56	−29.01, −12.10	< 0.001	< 0.001
Social functioning	541	1 vs. 0	−4.66	−11.56, 2.24	0.185	0.264
		≥ 2 vs. 0	−26.18	−35.43, −16.92	< 0.001	< 0.001
Role-emotional	538	1 vs. 0	3.78	−6.39, 13.96	0.465	0.465
		≥ 2 vs. 0	−11.03	−23.70, 1.64	0.088	0.088
Mental health	541	1 vs. 0	−5.38	−10.72, −0.03	0.049	0.081
		≥ 2 vs. 0	−16.29	−22.61, −9.96	< 0.001	< 0.001
Physical component summary	535	1 vs. 0	−4.25	−7.43, −1.08	0.009	0.029
		≥ 2 vs. 0	−12.82	−16.98, −8.66	< 0.001	< 0.001
Mental component summary	535	1 vs. 0	−1.51	−4.53, 1.51	0.327	0.364
		≥ 2 vs. 0	−6.10	−9.47, −2.73	< 0.001	< 0.001

^a^Models were adjusted for age, sex, race/ethnicity, educational attainment, employment status, and rurality. Respondents with unknown values for race/ethnicity, education, or employment were excluded; respondents with unknown rurality were retained. Analyses incorporated the final survey weight and stratified survey design. Benjamini-Hochberg false discovery rate correction was applied separately across the 10 HRQoL outcomes for each contrast

Compared with adults with no chronic conditions, adults with one condition had lower General Health scores (adjusted β = −11.09; 95% CI, − 18.10 to − 4.07; qFDR = 0.020), Vitality scores (adjusted β = −10.78; 95% CI, − 18.71 to − 2.84; qFDR = 0.029), and PCS scores (adjusted β = −4.25; 95% CI, − 7.43 to − 1.08; qFDR = 0.029) after FDR correction.

The largest adjusted differences among adults with two or more conditions were observed for Role-Physical (β = −30.65; 95% CI, − 46.56 to − 14.74), Physical Functioning (β = −28.70; 95% CI, − 38.60 to − 18.81), Bodily Pain (β = −28.28; 95% CI, − 38.96 to − 17.59), Social Functioning (β = −26.18; 95% CI, − 35.43 to − 16.92), and General Health (β = −24.18; 95% CI, − 30.75 to − 17.62), all with qFDR < 0.001.

Adults with two or more conditions also had lower Vitality (β = −20.56; 95% CI, − 29.01 to − 12.10), Mental Health (β = −16.29; 95% CI, − 22.61 to − 9.96), PCS (β = −12.82; 95% CI, − 16.98 to − 8.66), and MCS scores (β = −6.10; 95% CI, − 9.47 to − 2.73), all with qFDR < 0.001. The adjusted Role-Emotional difference was not statistically significant after FDR correction (β = −11.03; 95% CI, − 23.70 to 1.64; qFDR = 0.088) (Table 3; Fig. 1).

### HRQoL by specific chronic conditions

**Fig. 2:**
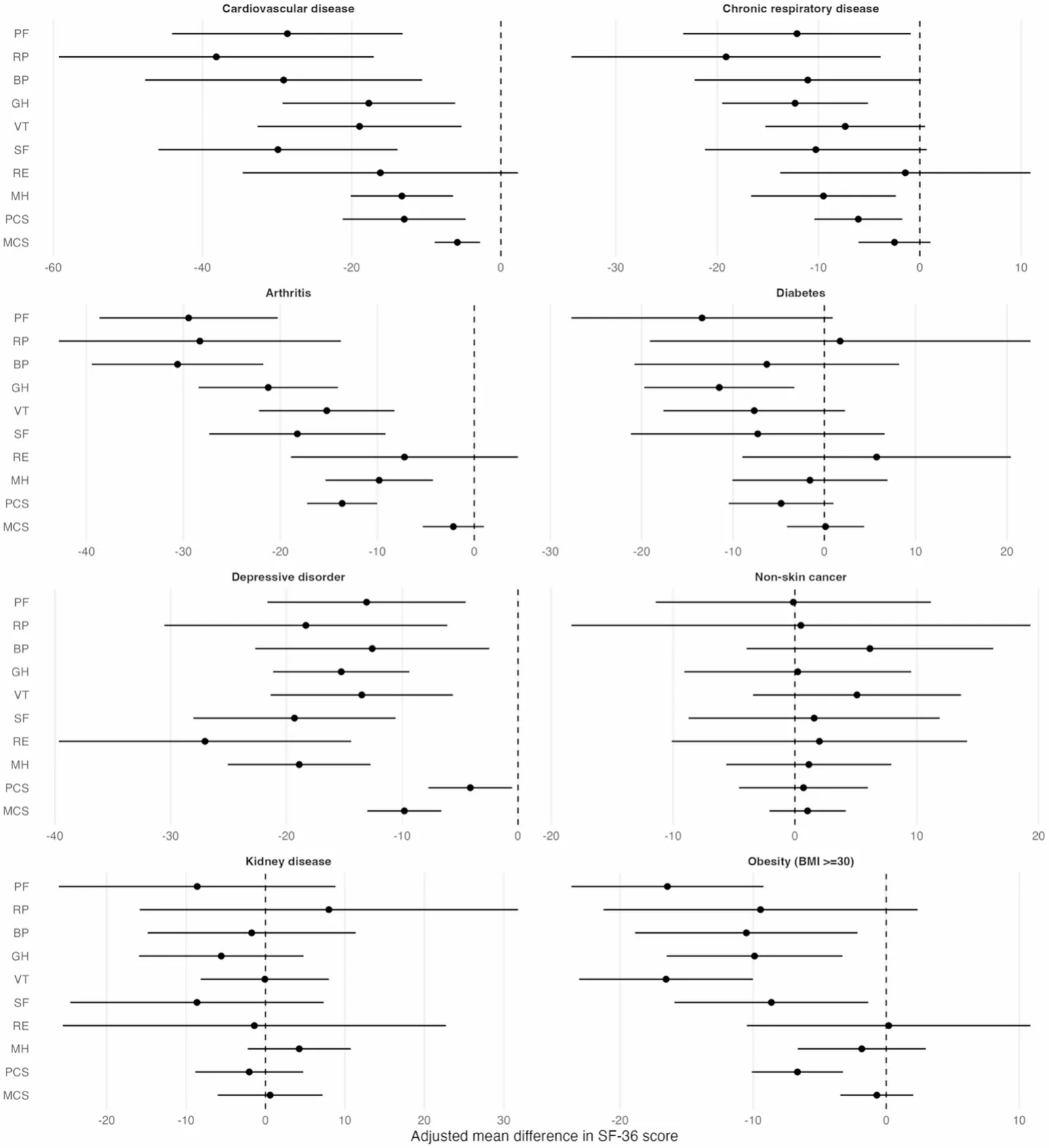
Adjusted associations between specific chronic conditions and SF-36 domain and summary scores. Points represent adjusted mean differences (β) and horizontal lines indicate 95% confidence intervals from survey-weighted linear regression models adjusted for age, sex, race/ethnicity, education, employment status, and rural/urban residence. Each panel corresponds to a specific chronic condition, with adults without the condition serving as the reference group. Negative values indicate lower health-related quality of life. PF, Physical Functioning; RP, Role-Physical; BP, Bodily Pain; GH, General Health; VT, Vitality; SF, Social Functioning; RE, Role-Emotional; MH, Mental Health; PCS, Physical Component Summary; MCS, Mental Component Summary

In condition-specific survey-weighted models, arthritis and cardiovascular disease showed the largest associations with lower PCS scores (Fig. 2; Supplementary Table S1). Arthritis was associated with a 13.63-point lower PCS score (95% CI, −17.25 to −10.01; qFDR < 0.001), and cardiovascular disease was associated with a 12.96-point lower PCS score (95% CI, −21.21 to −4.71; qFDR = 0.003).

Obesity, chronic respiratory disease, and depressive disorder were also associated with lower PCS scores after FDR correction. Obesity was associated with a 6.67-point lower PCS score (qFDR < 0.001), chronic respiratory disease with a 6.07-point lower score (qFDR = 0.031), and depressive disorder with a 4.12-point lower score (qFDR = 0.026). Diabetes, non-skin cancer, and kidney disease were not significantly associated with PCS after covariate adjustment and FDR correction (Supplementary Table S1).

For MCS, depressive disorder showed the largest adjusted difference (β = −9.81; 95% CI, − 13.01 to − 6.61; qFDR < 0.001), followed by cardiovascular disease (β = −5.85; 95% CI, −8.89 to −2.80; qFDR < 0.001). Chronic respiratory disease, arthritis, diabetes, non-skin cancer, kidney disease, and obesity were not associated with MCS after FDR correction (Supplementary Table S1).

Depressive disorder was associated with lower scores across all eight individual SF-36 domains. Associations were observed for Physical Functioning (β = −13.08; qFDR = 0.004), Role-Physical (β = −18.32; qFDR = 0.004), Bodily Pain (β = −12.59; qFDR = 0.016), General Health (β = −15.26; qFDR < 0.001), Vitality (β = −13.49; qFDR = 0.001), Social Functioning (β = −19.30; qFDR < 0.001), Role-Emotional (β = −27.03; qFDR < 0.001), and Mental Health (β = −18.89; qFDR < 0.001) (Supplementary Table S1).

### Sensitivity analyses

#### Treatment of unknown covariate values

In complete-case sensitivity analyses that additionally excluded the 28 respondents with unknown rurality, the adjusted associations comparing adults with two or more conditions with those with no conditions remained similar in magnitude to the primary estimates (Supplementary Table S2B). For example, the Physical Functioning coefficient was − 28.70 points in the primary analysis and − 29.38 points in the complete-case analysis. The corresponding coefficients were − 12.82 and − 13.18 points for PCS and − 6.10 and − 5.56 points for MCS. Role-Emotional remained the only outcome for which the two-or-more-versus-zero association was not statistically significant after FDR correction in either specification.

#### Flexible modeling of age

The associations were substantively stable when age was modeled with natural cubic splines of 3 or 4 degrees of freedom (Supplementary Table S2A; Supplementary Figure S1). For Physical Functioning, the adjusted two-or-more-versus-zero coefficient was − 28.70 points with linear age adjustment, compared with − 28.32 and − 28.67 points using three- and four-degree-of-freedom splines, respectively. The corresponding PCS coefficients were −12.82, −12.87, and − 12.96 points, and the corresponding MCS coefficients were − 6.10, − 6.55, and − 6.51 points. Flexible age adjustment therefore did not materially attenuate the primary estimates. Results were similarly stable across the remaining outcomes, although the Role-Emotional association became statistically significant under both spline specifications (qFDR = 0.037 for each specification). Exploratory age-stratified estimates are presented in Supplementary Table S7.

#### Depression-excluded chronic disease count

When depressive disorder was excluded from the chronic condition count, the association between two or more conditions and the Mental Health domain remained statistically significant (β = −12.90; 95% CI, −20.04 to −5.76; qFDR < 0.001). In contrast, the MCS association attenuated from −6.10 points in the primary analysis to −3.42 points (95% CI, −6.98 to 0.13; qFDR = 0.066) and was no longer statistically significant after FDR correction. In the more stringent model that excluded depression from the count and additionally adjusted for depressive disorder as a separate covariate, the Mental Health association remained significant (β = −8.56; 95% CI, −15.27 to −1.85; qFDR = 0.016), whereas the MCS association was further attenuated (β = −1.40; 95% CI, −4.72 to 1.93; qFDR = 0.456) (Supplementary Table S2C).

#### Full condition-count distribution and continuous-count models

The weighted mean number of conditions was 1.06 (SE, 0.10) overall and 2.69 (SE, 0.13) among adults with two or more conditions. The full uncollapsed distribution from zero to seven conditions is presented in Supplementary Table S3A. In continuous-count models, each additional condition was associated with lower scores across all 10 HRQoL outcomes (Supplementary Table S3B). Each additional condition was associated with an 11.05-point lower Physical Functioning score (95% CI, −14.18 to −7.92; qFDR < 0.001), a 4.57-point lower PCS score (95% CI, −6.13 to −3.02; qFDR < 0.001), and a 2.20-point lower MCS score (95% CI, −3.39 to −1.01; qFDR < 0.001). General Health showed evidence of departure from linearity (*p* = 0.036), whereas statistically significant departure from linearity was not detected for the other nine outcomes (Supplementary Table S3C).

#### Obesity-included and obesity-adjusted analyses

In sensitivity analyses using an eight-condition count that included obesity, 35.5% of the weighted population had no conditions, 22.6% had one condition, and 41.8% had two or more conditions. The associations between chronic disease burden and HRQoL remained substantial. The adjusted two-or-more-versus-zero coefficients were − 23.96 for Physical Functioning, −10.79 for PCS, and −4.32 for MCS under the obesity-included definition, compared with −28.70, −12.82, and −6.10, respectively, under the primary seven-condition definition (Supplementary Table S2C). Additional adjustment for obesity modestly attenuated several condition-specific associations but did not materially alter the principal conclusions. For arthritis, the adjusted Physical Functioning difference changed from − 29.46 points (95% CI, −38.66 to −20.26; qFDR < 0.001) to − 25.03 points (95% CI, − 33.93 to − 16.14; qFDR < 0.001) after adjustment for obesity. The corresponding PCS difference changed from − 13.63 to − 11.99 points, and both estimates remained statistically significant after FDR correction (Supplementary Table S4).

#### Exploratory rurality and race/ethnicity analyses

In exploratory analyses stratified by rurality, the weighted prevalence of multimorbidity was 36.3% among rural or nonmetro residents and 28.7% among urban or metro residents. Mean PCS scores were 44.2 among rural or nonmetro residents and 46.5 among urban or metro residents, whereas mean MCS scores were similar between the two groups, at 50.6 and 50.2, respectively (Supplementary Table S6A). There was no statistically significant evidence that the association between chronic disease burden and PCS or MCS differed by rurality (interaction *p* = 0.119 and *p* = 0.379, respectively; Supplementary Table S6C). In exploratory race/ethnicity interaction analyses, there was evidence that the association between chronic disease burden and PCS differed across the four race/ethnicity categories (omnibus interaction *p* = 0.002). The corresponding interaction for MCS was not statistically significant (*p* = 0.113) (Supplementary Tables S6B and C). These findings should be interpreted with caution because some race/ethnicity subgroups and chronic disease burden cells had few respondents.

## Discussion

In this survey-weighted study of Missouri adults, greater chronic disease burden was associated with lower HRQoL across nearly all SF-36 dimensions. The largest adjusted differences between adults with two or more conditions and those with no conditions were observed for Role-Physical, Physical Functioning, Bodily Pain, Social Functioning, and General Health. The PCS also declined markedly as chronic disease burden increased, whereas the association with the MCS was smaller. In the condition-specific analyses, arthritis and cardiovascular disease had the strongest associations with physical HRQoL, while depressive disorder had the strongest association with mental HRQoL. Our findings are consistent with previous research showing that HRQoL worsens as chronic conditions accumulate [1, 5, 12, 21–25], while providing a Missouri-specific view of which SF-36 domains are most affected and how the results change when depression and obesity are handled differently in sensitivity analyses.

The concentration of the largest differences in physical domains is consistent with, but extends, previous research. Fortin et al. [1] studied 238 Canadian primary care patients and incorporated both the number and severity of conditions across 14 organ-system domains. They found differences in the SF-36 PCS across 12 of the 14 morbidity domains, whereas the MCS differed primarily according to psychiatric morbidity. Similarly, Mujica-Mota et al. [12], using data from a large English national survey and the EQ-5D, found that HRQoL declined as the number of long-term conditions increased and that arthritis, neurological disorders, and long-term mental health problems were associated with particularly large reductions. A systematic review and meta-analysis by Makovski et al. [23] also found a progressive decline in HRQoL with each additional disease, with generally larger reductions in physical than mental HRQoL. More recently, a population-based study of U.S. adults aged 18–64 years found lower physical and mental HRQoL among adults with multimorbidity using the Veterans RAND-12 [24]. We examined all eight individual SF-36 domains alongside PCS and MCS across the full adult age range. This domain-level approach showed that the burden associated with multimorbidity involved not only physical functioning but also pain, limitations in usual physical roles, perceptions of general health, and social functioning.

The condition-specific results further clarify which conditions contributed most strongly to lower physical HRQoL. Arthritis and cardiovascular disease had the largest associations with lower PCS scores, and arthritis was strongly associated with Physical Functioning, Role-Physical, Bodily Pain, General Health, and Social Functioning. Fortin et al. [1] likewise found that severe musculoskeletal morbidity had the largest direct association with PCS and identified cardiac and respiratory morbidity as contributors to lower physical HRQoL. Mujica-Mota et al. [12] also identified arthritis and joint problems among the conditions associated with substantial HRQoL reductions. Longitudinal evidence has also linked musculoskeletal and cardiovascular conditions with declines in physical capability [10, 13, 14]. The present analysis adds evidence that the association between arthritis and physical HRQoL remained substantial after additional adjustment for obesity. The arthritis coefficient for Physical Functioning was attenuated from −29.46 to −25.03 points after obesity adjustment, but the association remained statistically significant after FDR correction.

The respiratory findings were less consistent than those for arthritis and cardiovascular disease. Chronic respiratory disease was associated with selected HRQoL outcomes, including Role-Physical, General Health, Mental Health, and PCS in the primary condition-specific analysis, although some associations were no longer statistically significant after additional adjustment for obesity and FDR correction. Separate exploratory analyses of asthma and COPD were imprecise, with asthma showing more consistent associations across physical domains than COPD. In comparison, arthritis and cardiovascular disease showed stronger and more consistent associations with physical HRQoL.

Depressive disorder was associated with lower scores across all eight SF-36 domains, including physical functioning, pain, general health, vitality, and social functioning. Previous studies have also shown that depression affects physical and social functioning in addition to mental health [15, 16, 26–28]. Mujica-Mota et al. [12] similarly found that adding a long-term mental health condition to existing physical conditions was associated with a larger reduction in HRQoL than adding another physical condition. In our sensitivity analyses, multimorbidity remained associated with the Mental Health domain after depressive disorder was removed from the condition count and entered separately as a covariate. The MCS association became smaller and was no longer statistically significant after FDR correction. Depression therefore appears to account for part of the MCS association, but not for the association between non-depression multimorbidity and the individual Mental Health domain. Both the number and type of conditions are important when interpreting mental HRQoL.

The adjusted differences were large relative to the variability observed among adults with no chronic conditions. The differences in Physical Functioning and Role-Physical corresponded to approximately 1.87 and 1.02 standard deviations, respectively, and the differences in Bodily Pain, General Health, Social Functioning, and PCS ranged from 1.53 to 1.72 standard deviations (Supplementary Table S8). Methodological studies have used approximately one-half standard deviation as a general reference when interpreting differences in HRQoL measures [29–31]. This value does not represent a universal minimally important difference for every SF-36 domain. Because the study was cross-sectional, the estimates describe differences between chronic disease burden groups rather than clinically important changes within individuals over time.

The continuous-count analysis showed a clear gradient, with scores declining across all 10 HRQoL outcomes as the number of conditions increased, including PCS and MCS. Similar patterns have been reported in primary care studies and population surveys [12, 21–24]. The per-condition coefficient should be interpreted as an average across the observed count range, because General Health showed evidence of nonlinearity and few respondents had five or more conditions. The data therefore do not support assuming that every additional condition produces the same change in HRQoL at each point in the count.

Results changed little when age was modeled with natural cubic splines using three or four degrees of freedom or when respondents with unknown rurality were excluded in the complete-case analysis. Estimates for Physical Functioning, PCS, MCS, and most other outcomes remained close to those from the primary models. Role-Emotional was more sensitive to the age specification, with the association reaching statistical significance in both spline models. In the age-stratified analyses, multimorbidity was associated with poorer physical HRQoL among adults aged 18–44, 45–64, and 65 years or older. Although multimorbidity was most common in the oldest group, it was also present among younger adults, as reported in previous studies showing that multimorbidity can develop before older age, particularly in socially disadvantaged populations [32, 33]. Because these subgroup analyses were exploratory, they should not be interpreted as a formal test of whether the association differed by age.

Obesity was not included in the primary seven-condition count because it was based on BMI calculated from self-reported height and weight, while the other conditions were identified through diagnosis questions. Adding obesity increased the weighted prevalence of multimorbidity from 30.0% to 41.8% and reduced the size of the associations with Physical Functioning, PCS, and MCS, although the estimates remained substantial. The change in prevalence and effect size reflects how strongly multimorbidity estimates depend on which conditions are included [9, 11]. Accordingly, our 30.0% estimate should not be compared directly with surveillance estimates based on broader condition lists or different methods of identifying chronic conditions. Obesity is also difficult to classify in this relationship because it may precede chronic disease, contribute to functional limitation, or develop after activity becomes restricted.

In the exploratory analyses, rural or nonmetro adults had a higher weighted prevalence of multimorbidity and a lower mean PCS score than urban or metro adults, but the confidence intervals were wide, and the rurality interaction was not significant for either PCS or MCS. The four-category race/ethnicity interaction was significant only for PCS, and several subgroup cells were small, limiting the precision of those estimates. Education, income, employment, health care access, and neighborhood conditions may also contribute to differences in HRQoL across these groups [18, 19, 34].

A condition count can identify adults with poorer HRQoL, but it does not show which parts of daily life are most affected or which conditions are most closely related to those differences. In this study, arthritis and cardiovascular disease were most strongly associated with mobility, pain, endurance, and limitations in usual physical roles, while depressive disorder was associated with physical, social, and mental domains. Looking at individual HRQoL domains therefore provides information that is lost when only the number of diagnoses is considered. Evidence from multimorbidity care models remains mixed, partly because patients have very different combinations of conditions and interventions vary in their focus and intensity [35]. The overlap between physical and mental health also supports care that addresses both together [36, 37].

This study has several strengths. The use of the SF-36 enabled examination of eight individual HRQoL domains and both component summaries, allowing physical, role, pain, social, and mental dimensions to be distinguished. The study included adults across a broad age range and used survey weights to produce estimates relevant to the Missouri adult population. The analysis evaluated both overall chronic disease burden and individual conditions within the same framework. Multiple sensitivity analyses examined the treatment of unknown covariate values, the functional form of age, the exclusion of depression, the inclusion and adjustment of obesity, and the full uncollapsed condition count.

Several limitations warrant consideration. First, the cross-sectional design precludes conclusions about temporal sequence or causality. Chronic conditions may reduce HRQoL, but lower functioning and related behaviors may also contribute to the development or recognition of chronic conditions. Second, chronic conditions, height, weight, and HRQoL were self-reported, introducing the possibility of recall, reporting, and classification errors. The diagnosis-based items may also underestimate undiagnosed disease. Third, institutionalized adults were excluded by design, and adults with severe illness who were otherwise eligible may have been less likely to participate, potentially leading to underrepresentation of adults with greater functional impairment and more favorable HRQoL estimates. Survey weighting reduces but cannot eliminate nonresponse, selection, or coverage bias.

Educational attainment in the weighted sample was higher than the corresponding Missouri population benchmark. Overall, 39.8% of the weighted sample had a bachelor’s degree or higher. In an age-comparable analysis restricted to adults aged 25 years or older, 44.3% had a bachelor’s degree or higher, compared with 32.4% of Missouri residents aged 25 years or older in the 2020–2024 U.S. Census Bureau estimates [25]. Adults with higher educational attainment may therefore remain overrepresented despite weighting. Because education is associated with better health and HRQoL, the descriptive SF-36 means may be somewhat more favorable than those of the broader Missouri population. Education was included in the adjusted models, but this adjustment cannot fully address selection or coverage bias related to the educational composition of the sample. The absolute HRQoL estimates should therefore be generalized to the Missouri population with caution.

Ceiling effects in the Role-Physical and Role-Emotional domains may have reduced the ability to distinguish respondents with fewer conditions and may partly explain the less consistent Role-Emotional findings. Our sample size also reduced precision in the condition-specific, age-stratified, rurality, and race/ethnicity analyses. In addition, the primary burden measure was a simple condition count that gave equal weight to conditions differing in severity, duration, treatment, and functional consequences and did not account for specific disease combinations. Severity-weighted measures and disease-pattern approaches may capture information that is not reflected in a simple count [1, 10, 12, 21].

## Conclusion

Among Missouri adults, a greater burden of chronic conditions was associated with lower HRQoL across most SF-36 domains, with the largest differences in physical functioning, physical role limitations, pain, general health, social functioning, and PCS. Arthritis and cardiovascular disease had the strongest associations with physical HRQoL, while depressive disorder had the strongest association with mental HRQoL. Sensitivity analyses showed that the MCS association became smaller when depressive disorder was removed from the condition count and included as a separate covariate. Adults with a similar number of conditions may still differ considerably in mobility, pain, daily activities, social participation, and mental well-being, so incorporating HRQoL measures into chronic disease surveillance and clinical follow-up in Missouri may help identify needs that are not evident from diagnoses alone and support more targeted care and local health planning. Because this study included only Missouri adults, the estimates may not be generalizable to populations in other states.

## Supplementary Information

Below is the link to the electronic supplementary material.

Supplementary Material 1

Supplementary Material 2

## Data Availability

The data analyzed in this study are from the 2024 Center for Medical Epidemiology and Population Health Adult Health Survey. The data are not publicly available because they were collected under University of Missouri institutional protocols and may contain sensitive survey information. Deidentified data may be available from the corresponding author upon reasonable request and with permission from the Center for Medical Epidemiology and Population Health and applicable institutional approvals.
