# Supplementary material for: Chronic disease burden and health-related quality of life among missouri adults
Source: Qual Life Res. 2026 Nov 1;35(11):318. doi: 10.1007/s11136-026-04422-6 (PMC13724639; doi:10.1007/s11136-026-04422-6)
Supplement: Supplementary file 1 [file 11136_2026_4422_MOESM1_ESM.docx]

**Supplementary Tables**

# **Supplementary Table S1.** Adjusted associations between individual chronic conditions and SF-36 outcomes

| **Condition** | **SF-36 outcome** | **Analytic n** | **Adjusted β (95% CI)** | **qFDR** |
| --- | --- | --- | --- | --- |
| Cardiovascular disease | Physical Functioning | 541 | −28.62 (−44.07 to −13.16) | <0.001 |
| Cardiovascular disease | Role-Physical | 538 | −38.15 (−59.25 to −17.05) | <0.001 |
| Cardiovascular disease | Bodily Pain | 540 | −29.12 (−47.69 to −10.55) | 0.003 |
| Cardiovascular disease | General Health | 541 | −17.71 (−29.28 to −6.14) | 0.003 |
| Cardiovascular disease | Vitality | 541 | −18.95 (−32.62 to −5.28) | 0.007 |
| Cardiovascular disease | Social Functioning | 541 | −29.89 (−45.91 to −13.86) | <0.001 |
| Cardiovascular disease | Role-Emotional | 538 | −16.16 (−34.61 to 2.28) | 0.086 |
| Cardiovascular disease | Mental Health | 541 | −13.28 (−20.15 to −6.40) | <0.001 |
| Cardiovascular disease | Physical Component Summary | 535 | −12.96 (−21.21 to −4.71) | 0.003 |
| Cardiovascular disease | Mental Component Summary | 535 | −5.85 (−8.89 to −2.80) | <0.001 |
| Chronic respiratory disease | Physical Functioning | 541 | −12.12 (−23.35 to −0.89) | 0.069 |
| Chronic respiratory disease | Role-Physical | 538 | −19.12 (−34.38 to −3.85) | 0.036 |
| Chronic respiratory disease | Bodily Pain | 540 | −11.06 (−22.23 to 0.11) | 0.084 |
| Chronic respiratory disease | General Health | 541 | −12.31 (−19.51 to −5.11) | 0.008 |
| Chronic respiratory disease | Vitality | 541 | −7.36 (−15.25 to 0.52) | 0.084 |
| Chronic respiratory disease | Social Functioning | 541 | −10.26 (−21.20 to 0.68) | 0.084 |
| Chronic respiratory disease | Role-Emotional | 538 | −1.44 (−13.79 to 10.91) | 0.819 |
| Chronic respiratory disease | Mental Health | 541 | −9.51 (−16.66 to −2.37) | 0.031 |
| Chronic respiratory disease | Physical Component Summary | 535 | −6.07 (−10.41 to −1.73) | 0.031 |
| Chronic respiratory disease | Mental Component Summary | 535 | −2.50 (−6.06 to 1.05) | 0.186 |
| Arthritis | Physical Functioning | 536 | −29.46 (−38.66 to −20.26) | <0.001 |
| Arthritis | Role-Physical | 533 | −28.30 (−42.85 to −13.75) | <0.001 |
| Arthritis | Bodily Pain | 535 | −30.60 (−39.45 to −21.75) | <0.001 |
| Arthritis | General Health | 536 | −21.25 (−28.44 to −14.06) | <0.001 |
| Arthritis | Vitality | 536 | −15.21 (−22.20 to −8.22) | <0.001 |
| Arthritis | Social Functioning | 536 | −18.25 (−27.34 to −9.15) | <0.001 |
| Arthritis | Role-Emotional | 533 | −7.20 (−18.91 to 4.51) | 0.228 |
| Arthritis | Mental Health | 536 | −9.81 (−15.35 to −4.26) | <0.001 |
| Arthritis | Physical Component Summary | 530 | −13.63 (−17.25 to −10.01) | <0.001 |
| Arthritis | Mental Component Summary | 530 | −2.16 (−5.31 to 0.99) | 0.199 |
| Diabetes | Physical Functioning | 537 | −13.39 (−27.70 to 0.92) | 0.326 |
| Diabetes | Role-Physical | 534 | 1.74 (−19.10 to 22.57) | 0.951 |
| Diabetes | Bodily Pain | 536 | −6.30 (−20.79 to 8.19) | 0.635 |
| Diabetes | General Health | 537 | −11.50 (−19.71 to −3.29) | 0.062 |
| Diabetes | Vitality | 537 | −7.67 (−17.62 to 2.27) | 0.326 |
| Diabetes | Social Functioning | 537 | −7.29 (−21.18 to 6.61) | 0.607 |
| Diabetes | Role-Emotional | 534 | 5.72 (−8.98 to 20.43) | 0.635 |
| Diabetes | Mental Health | 537 | −1.57 (−10.07 to 6.92) | 0.895 |
| Diabetes | Physical Component Summary | 531 | −4.73 (−10.47 to 1.01) | 0.326 |
| Diabetes | Mental Component Summary | 531 | 0.13 (−4.09 to 4.36) | 0.951 |
| Depressive disorder | Physical Functioning | 533 | −13.08 (−21.65 to −4.51) | 0.004 |
| Depressive disorder | Role-Physical | 530 | −18.32 (−30.54 to −6.10) | 0.004 |
| Depressive disorder | Bodily Pain | 532 | −12.59 (−22.69 to −2.48) | 0.016 |
| Depressive disorder | General Health | 533 | −15.26 (−21.15 to −9.37) | <0.001 |
| Depressive disorder | Vitality | 533 | −13.49 (−21.37 to −5.61) | 0.001 |
| Depressive disorder | Social Functioning | 533 | −19.30 (−28.04 to −10.57) | <0.001 |
| Depressive disorder | Role-Emotional | 530 | −27.03 (−39.66 to −14.41) | <0.001 |
| Depressive disorder | Mental Health | 533 | −18.89 (−25.06 to −12.73) | <0.001 |
| Depressive disorder | Physical Component Summary | 527 | −4.12 (−7.73 to −0.50) | 0.026 |
| Depressive disorder | Mental Component Summary | 527 | −9.81 (−13.01 to −6.61) | <0.001 |
| Non-skin cancer | Physical Functioning | 536 | −0.13 (−11.42 to 11.16) | 0.982 |
| Non-skin cancer | Role-Physical | 533 | 0.49 (−18.35 to 19.34) | 0.982 |
| Non-skin cancer | Bodily Pain | 535 | 6.16 (−3.97 to 16.28) | 0.982 |
| Non-skin cancer | General Health | 536 | 0.23 (−9.08 to 9.55) | 0.982 |
| Non-skin cancer | Vitality | 536 | 5.10 (−3.44 to 13.63) | 0.982 |
| Non-skin cancer | Social Functioning | 536 | 1.58 (−8.73 to 11.89) | 0.982 |
| Non-skin cancer | Role-Emotional | 533 | 2.01 (−10.11 to 14.14) | 0.982 |
| Non-skin cancer | Mental Health | 536 | 1.15 (−5.63 to 7.92) | 0.982 |
| Non-skin cancer | Physical Component Summary | 530 | 0.71 (−4.59 to 6.00) | 0.982 |
| Non-skin cancer | Mental Component Summary | 530 | 1.04 (−2.09 to 4.17) | 0.982 |
| Kidney disease | Physical Functioning | 537 | −8.60 (−26.02 to 8.82) | 0.831 |
| Kidney disease | Role-Physical | 534 | 7.98 (−15.83 to 31.78) | 0.925 |
| Kidney disease | Bodily Pain | 536 | −1.74 (−14.83 to 11.36) | 0.984 |
| Kidney disease | General Health | 537 | −5.57 (−15.93 to 4.80) | 0.831 |
| Kidney disease | Vitality | 537 | −0.08 (−8.16 to 7.99) | 0.984 |
| Kidney disease | Social Functioning | 537 | −8.63 (−24.59 to 7.33) | 0.831 |
| Kidney disease | Role-Emotional | 534 | −1.41 (−25.52 to 22.71) | 0.984 |
| Kidney disease | Mental Health | 537 | 4.25 (−2.25 to 10.74) | 0.831 |
| Kidney disease | Physical Component Summary | 531 | −2.05 (−8.85 to 4.76) | 0.925 |
| Kidney disease | Mental Component Summary | 531 | 0.58 (−6.03 to 7.20) | 0.984 |
| Obesity (BMI ≥30 kg/m²) | Physical Functioning | 518 | −16.44 (−23.66 to −9.22) | <0.001 |
| Obesity (BMI ≥30 kg/m²) | Role-Physical | 515 | −9.45 (−21.25 to 2.35) | 0.166 |
| Obesity (BMI ≥30 kg/m²) | Bodily Pain | 517 | −10.51 (−18.87 to −2.16) | 0.028 |
| Obesity (BMI ≥30 kg/m²) | General Health | 518 | −9.89 (−16.50 to −3.28) | 0.009 |
| Obesity (BMI ≥30 kg/m²) | Vitality | 518 | −16.54 (−23.07 to −10.01) | <0.001 |
| Obesity (BMI ≥30 kg/m²) | Social Functioning | 518 | −8.63 (−15.91 to −1.35) | 0.034 |
| Obesity (BMI ≥30 kg/m²) | Role-Emotional | 515 | 0.18 (−10.47 to 10.84) | 0.973 |
| Obesity (BMI ≥30 kg/m²) | Mental Health | 518 | −1.84 (−6.66 to 2.97) | 0.566 |
| Obesity (BMI ≥30 kg/m²) | Physical Component Summary | 512 | −6.67 (−10.10 to −3.24) | <0.001 |
| Obesity (BMI ≥30 kg/m²) | Mental Component Summary | 512 | −0.71 (−3.45 to 2.04) | 0.681 |

***Note.*** *β is the survey-weighted adjusted mean difference comparing respondents with versus without the listed condition. Negative estimates indicate lower HRQoL among respondents with the condition. Models adjusted for age, sex, race/ethnicity, educational attainment, employment status, and rurality. Respondents with sparse unknown values for race/ethnicity, education, or employment were excluded; unknown rurality was retained. qFDR was calculated across the 10 HRQoL outcomes separately within each condition.*

# **Supplementary Table S2A.** Sensitivity of the adjusted ≥2-versus-0 chronic disease burden contrast to alternative age specifications

| **SF-36 outcome** | **n** | **Linear age β (95% CI)** | **qFDR** | **Natural cubic spline, 3 df β (95% CI)** | **qFDR** | **Natural cubic spline, 4 df β (95% CI)** | **qFDR** |
| --- | --- | --- | --- | --- | --- | --- | --- |
| Physical Functioning | 541 | −28.70 (−38.60 to −18.81) | <0.001 | −28.32 (−38.00 to −18.64) | <0.001 | −28.67 (−38.16 to −19.17) | <0.001 |
| Role-Physical | 538 | −30.65 (−46.56 to −14.74) | <0.001 | −32.59 (−47.72 to −17.46) | <0.001 | −32.57 (−47.88 to −17.26) | <0.001 |
| Bodily Pain | 540 | −28.28 (−38.96 to −17.59) | <0.001 | −27.92 (−37.74 to −18.10) | <0.001 | −28.05 (−37.83 to −18.26) | <0.001 |
| General Health | 541 | −24.18 (−30.75 to −17.62) | <0.001 | −24.48 (−30.76 to −18.19) | <0.001 | −24.59 (−30.84 to −18.34) | <0.001 |
| Vitality | 541 | −20.56 (−29.01 to −12.10) | <0.001 | −22.21 (−30.04 to −14.37) | <0.001 | −22.26 (−30.14 to −14.37) | <0.001 |
| Social Functioning | 541 | −26.18 (−35.43 to −16.92) | <0.001 | −26.63 (−35.05 to −18.21) | <0.001 | −26.66 (−35.18 to −18.15) | <0.001 |
| Role-Emotional | 538 | −11.03 (−23.70 to 1.64) | 0.088 | −12.94 (−25.10 to −0.78) | 0.037 | −13.05 (−25.32 to −0.78) | 0.037 |
| Mental Health | 541 | −16.29 (−22.61 to −9.96) | <0.001 | −16.10 (−22.27 to −9.94) | <0.001 | −16.02 (−22.26 to −9.79) | <0.001 |
| Physical Component Summary | 535 | −12.82 (−16.98 to −8.66) | <0.001 | −12.87 (−16.68 to −9.06) | <0.001 | −12.96 (−16.75 to −9.18) | <0.001 |
| Mental Component Summary | 535 | −6.10 (−9.47 to −2.73) | <0.001 | −6.55 (−9.78 to −3.32) | <0.001 | −6.51 (−9.79 to −3.23) | <0.001 |

***Note.*** *All models adjusted for sex, race/ethnicity, education, employment, and rurality. The spline models used natural cubic splines with three and four degrees of freedom. qFDR was calculated across the 10 outcomes within the ≥2-versus-0 contrast.*

# **Supplementary Table S2B.** Sensitivity of the adjusted ≥2-versus-0 association to the treatment of unknown covariate values

| **SF-36 outcome** | **Primary n** | **Primary β (95% CI)** | **qFDR** | **Complete-case n** | **Complete-case β (95% CI)** | **qFDR** |
| --- | --- | --- | --- | --- | --- | --- |
| Physical Functioning | 541 | −28.70 (−38.60 to −18.81) | <0.001 | 513 | −29.38 (−39.82 to −18.94) | <0.001 |
| Role-Physical | 538 | −30.65 (−46.56 to −14.74) | <0.001 | 510 | −30.94 (−47.98 to −13.90) | <0.001 |
| Bodily Pain | 540 | −28.28 (−38.96 to −17.59) | <0.001 | 513 | −28.85 (−39.97 to −17.73) | <0.001 |
| General Health | 541 | −24.18 (−30.75 to −17.62) | <0.001 | 513 | −24.05 (−31.07 to −17.02) | <0.001 |
| Vitality | 541 | −20.56 (−29.01 to −12.10) | <0.001 | 513 | −20.41 (−29.44 to −11.39) | <0.001 |
| Social Functioning | 541 | −26.18 (−35.43 to −16.92) | <0.001 | 513 | −25.82 (−35.70 to −15.93) | <0.001 |
| Role-Emotional | 538 | −11.03 (−23.70 to 1.64) | 0.088 | 511 | −9.36 (−22.59 to 3.88) | 0.166 |
| Mental Health | 541 | −16.29 (−22.61 to −9.96) | <0.001 | 513 | −15.84 (−22.45 to −9.22) | <0.001 |
| Physical Component Summary | 535 | −12.82 (−16.98 to −8.66) | <0.001 | 508 | −13.18 (−17.58 to −8.78) | <0.001 |
| Mental Component Summary | 535 | −6.10 (−9.47 to −2.73) | <0.001 | 508 | −5.56 (−9.10 to −2.03) | 0.002 |

***Note.*** *The primary models excluded six respondents with unknown race/ethnicity, education, or employment and retained unknown rurality. The complete-case models additionally excluded 28 respondents with unknown rurality.*

# **Supplementary Table S2C.** Sensitivity to alternative chronic condition definitions

## **Panel A.** Depression-excluded condition count

| **SF-36 outcome** | **Primary 7-condition count β (95% CI)** | **qFDR** | **Depression-excluded count β (95% CI)** | **qFDR** | **Depression-excluded count + depression covariate β (95% CI)** | **qFDR** |
| --- | --- | --- | --- | --- | --- | --- |
| Physical Functioning | −28.70 (−38.60 to −18.81) | <0.001 | −33.41 (−44.34 to −22.47) | <0.001 | −33.60 (−44.28 to −22.91) | <0.001 |
| Role-Physical | −30.65 (−46.56 to −14.74) | <0.001 | −33.10 (−51.49 to −14.72) | <0.001 | −29.73 (−48.92 to −10.54) | 0.004 |
| Bodily Pain | −28.28 (−38.96 to −17.59) | <0.001 | −32.80 (−45.23 to −20.36) | <0.001 | −30.57 (−44.25 to −16.88) | <0.001 |
| General Health | −24.18 (−30.75 to −17.62) | <0.001 | −25.16 (−32.85 to −17.48) | <0.001 | −22.91 (−30.93 to −14.90) | <0.001 |
| Vitality | −20.56 (−29.01 to −12.10) | <0.001 | −18.50 (−28.08 to −8.92) | <0.001 | −16.96 (−27.13 to −6.80) | 0.002 |
| Social Functioning | −26.18 (−35.43 to −16.92) | <0.001 | −25.43 (−36.19 to −14.67) | <0.001 | −23.24 (−34.63 to −11.85) | <0.001 |
| Role-Emotional | −11.03 (−23.70 to 1.64) | 0.088 | −5.58 (−19.07 to 7.90) | 0.416 | −0.70 (−13.11 to 11.72) | 0.912 |
| Mental Health | −16.29 (−22.61 to −9.96) | <0.001 | −12.90 (−20.04 to −5.76) | <0.001 | −8.56 (−15.27 to −1.85) | 0.016 |
| Physical Component Summary | −12.82 (−16.98 to −8.66) | <0.001 | −15.39 (−20.07 to −10.72) | <0.001 | −15.30 (−20.31 to −10.30) | <0.001 |
| Mental Component Summary | −6.10 (−9.47 to −2.73) | <0.001 | −3.42 (−6.98 to 0.13) | 0.066 | −1.40 (−4.72 to 1.93) | 0.456 |

## **Panel B.** Obesity-included condition count

| **SF-36 outcome** | **Primary 7-condition count β (95% CI)** | **qFDR** | **Obesity-included 8-condition count β (95% CI)** | **qFDR** |
| --- | --- | --- | --- | --- |
| Physical Functioning | −28.70 (−38.60 to −18.81) | <0.001 | −23.96 (−32.00 to −15.92) | <0.001 |
| Role-Physical | −30.65 (−46.56 to −14.74) | <0.001 | −23.59 (−37.37 to −9.80) | 0.001 |
| Bodily Pain | −28.28 (−38.96 to −17.59) | <0.001 | −21.95 (−31.66 to −12.25) | <0.001 |
| General Health | −24.18 (−30.75 to −17.62) | <0.001 | −21.01 (−27.33 to −14.68) | <0.001 |
| Vitality | −20.56 (−29.01 to −12.10) | <0.001 | −21.06 (−28.25 to −13.86) | <0.001 |
| Social Functioning | −26.18 (−35.43 to −16.92) | <0.001 | −18.54 (−26.36 to −10.72) | <0.001 |
| Role-Emotional | −11.03 (−23.70 to 1.64) | 0.088 | −7.44 (−18.94 to 4.07) | 0.205 |
| Mental Health | −16.29 (−22.61 to −9.96) | <0.001 | −11.46 (−16.90 to −6.01) | <0.001 |
| Physical Component Summary | −12.82 (−16.98 to −8.66) | <0.001 | −10.79 (−14.26 to −7.32) | <0.001 |
| Mental Component Summary | −6.10 (−9.47 to −2.73) | <0.001 | −4.32 (−7.30 to −1.33) | 0.005 |

***Note.*** *All estimates compare adults with two or more conditions to adults with no conditions, using the specified count definition. Models used the same covariate adjustment and FDR procedure as the primary analysis.*

### **Panel C.** Distribution of chronic disease burden after inclusion of obesity

| **Chronic disease burden** | **Unweighted n** | **Unweighted %** | **Weighted % (95% CI)** |
| --- | --- | --- | --- |
| 0 conditions | 142 | 26.0 | 35.5 (27.6 to 43.4) |
| 1 condition | 138 | 25.2 | 22.6 (16.0 to 29.2) |
| ≥2 conditions | 267 | 48.8 | 41.8 (34.0 to 49.6) |

***Note.*** *The obesity included count comprised the seven primary chronic condition groups plus obesity, defined as body mass index ≥30 kg/m². Weighted percentages and 95% confidence intervals incorporated the survey weights and stratified survey design.*

# **Supplementary Table S3A.** Full distribution of the seven-condition count

| **Number of conditions** | **Unweighted n** | **Unweighted %** | **Weighted % (95% CI)** |
| --- | --- | --- | --- |
| 0 | 184 | 33.6 | 45.1 (37.0 to 53.2) |
| 1 | 155 | 28.3 | 24.8 (18.4 to 31.3) |
| 2 | 110 | 20.1 | 16.6 (10.8 to 22.4) |
| 3 | 56 | 10.2 | 7.8 (3.9 to 11.8) |
| 4 | 28 | 5.1 | 4.1 (1.3 to 7.0) |
| 5 | 13 | 2.4 | 1.3 (0.0 to 3.2) |
| 6 | 1 | 0.2 | 0.2 (0.0 to 0.6) |
| 7 | 0 | 0.0 | 0.0 (0.0 to 0.0) |

**Weighted mean count:** 1.06 (SE, 0.10) overall and 2.69 (SE, 0.13) among adults with two or more conditions.

# **Supplementary Table S3B.** Continuous condition-count associations with SF-36 outcomes

| **SF-36 outcome** | **Analytic n** | **Adjusted β per additional condition (95% CI)** | **qFDR** |
| --- | --- | --- | --- |
| Physical Functioning | 541 | −11.05 (−14.18 to −7.92) | <0.001 |
| Role-Physical | 538 | −10.66 (−17.16 to −4.16) | 0.002 |
| Bodily Pain | 540 | −9.46 (−13.22 to −5.70) | <0.001 |
| General Health | 541 | −9.04 (−11.51 to −6.56) | <0.001 |
| Vitality | 541 | −6.44 (−9.77 to −3.11) | <0.001 |
| Social Functioning | 541 | −9.42 (−12.99 to −5.85) | <0.001 |
| Role-Emotional | 538 | −5.47 (−9.55 to −1.39) | 0.009 |
| Mental Health | 541 | −5.72 (−8.07 to −3.37) | <0.001 |
| Physical Component Summary | 535 | −4.57 (−6.13 to −3.02) | <0.001 |
| Mental Component Summary | 535 | −2.20 (−3.39 to −1.01) | <0.001 |

***Note.*** *Negative coefficients indicate lower HRQoL associated with each additional condition. Models adjusted for age, sex, race/ethnicity, education, employment, and rurality.*

# **Supplementary Table S3C.** Design-based tests of departure from linearity for the condition count

| **SF-36 outcome** | **Analytic n** | **Test df** | **p for departure from linearity** |
| --- | --- | --- | --- |
| Physical Functioning | 541 | 4 | 0.077 |
| Role-Physical | 538 | 4 | 0.197 |
| Bodily Pain | 540 | 4 | 0.084 |
| General Health | 541 | 4 | 0.036 |
| Vitality | 541 | 4 | 0.118 |
| Social Functioning | 541 | 4 | 0.140 |
| Role-Emotional | 538 | 4 | 0.239 |
| Mental Health | 541 | 4 | 0.285 |
| Physical Component Summary | 535 | 4 | 0.072 |
| Mental Component Summary | 535 | 4 | 0.827 |

***Note.*** *The continuous condition-count specification was evaluated against a categorical specification of 0, 1, 2, 3, and 4 or more conditions using a design-based Wald test.*

# **Supplementary Table S4.** Condition-specific associations after additional adjustment for obesity

| **Condition** | **SF-36 outcome** | **Analytic n** | **Obesity-adjusted β (95% CI)** | **qFDR** |
| --- | --- | --- | --- | --- |
| Cardiovascular disease | Physical Functioning | 518 | −26.78 (−40.08 to −13.49) | <0.001 |
| Cardiovascular disease | Role-Physical | 515 | −37.12 (−57.67 to −16.57) | <0.001 |
| Cardiovascular disease | Bodily Pain | 517 | −27.30 (−44.51 to −10.10) | 0.002 |
| Cardiovascular disease | General Health | 518 | −16.82 (−27.37 to −6.27) | 0.002 |
| Cardiovascular disease | Vitality | 518 | −17.83 (−28.46 to −7.19) | 0.002 |
| Cardiovascular disease | Social Functioning | 518 | −28.72 (−43.52 to −13.92) | <0.001 |
| Cardiovascular disease | Role-Emotional | 515 | −15.75 (−34.23 to 2.74) | 0.095 |
| Cardiovascular disease | Mental Health | 518 | −13.13 (−20.04 to −6.23) | <0.001 |
| Cardiovascular disease | Physical Component Summary | 512 | −12.17 (−19.44 to −4.91) | 0.002 |
| Cardiovascular disease | Mental Component Summary | 512 | −5.81 (−8.98 to −2.63) | <0.001 |
| Chronic respiratory disease | Physical Functioning | 518 | −10.34 (−21.54 to 0.87) | 0.141 |
| Chronic respiratory disease | Role-Physical | 515 | −17.89 (−33.80 to −1.99) | 0.069 |
| Chronic respiratory disease | Bodily Pain | 517 | −8.92 (−20.57 to 2.74) | 0.191 |
| Chronic respiratory disease | General Health | 518 | −11.36 (−18.99 to −3.73) | 0.036 |
| Chronic respiratory disease | Vitality | 518 | −5.67 (−13.72 to 2.38) | 0.209 |
| Chronic respiratory disease | Social Functioning | 518 | −8.83 (−19.92 to 2.26) | 0.191 |
| Chronic respiratory disease | Role-Emotional | 515 | −1.01 (−13.40 to 11.38) | 0.873 |
| Chronic respiratory disease | Mental Health | 518 | −9.08 (−16.28 to −1.88) | 0.068 |
| Chronic respiratory disease | Physical Component Summary | 512 | −5.24 (−9.89 to −0.60) | 0.069 |
| Chronic respiratory disease | Mental Component Summary | 512 | −2.31 (−5.87 to 1.25) | 0.226 |
| Arthritis | Physical Functioning | 513 | −25.03 (−33.93 to −16.14) | <0.001 |
| Arthritis | Role-Physical | 510 | −25.22 (−39.54 to −10.90) | <0.001 |
| Arthritis | Bodily Pain | 512 | −28.27 (−36.84 to −19.69) | <0.001 |
| Arthritis | General Health | 513 | −19.49 (−26.30 to −12.69) | <0.001 |
| Arthritis | Vitality | 513 | −11.85 (−18.65 to −5.05) | <0.001 |
| Arthritis | Social Functioning | 513 | −16.52 (−25.53 to −7.51) | <0.001 |
| Arthritis | Role-Emotional | 510 | −5.55 (−18.31 to 7.22) | 0.394 |
| Arthritis | Mental Health | 513 | −9.80 (−15.40 to −4.20) | <0.001 |
| Arthritis | Physical Component Summary | 507 | −11.99 (−15.46 to −8.52) | <0.001 |
| Arthritis | Mental Component Summary | 507 | −2.08 (−5.43 to 1.27) | 0.247 |
| Diabetes | Physical Functioning | 514 | −11.16 (−24.92 to 2.60) | 0.559 |
| Diabetes | Role-Physical | 511 | 4.25 (−18.03 to 26.53) | 0.867 |
| Diabetes | Bodily Pain | 513 | −5.15 (−20.31 to 10.01) | 0.837 |
| Diabetes | General Health | 514 | −9.15 (−18.26 to −0.03) | 0.493 |
| Diabetes | Vitality | 514 | −3.77 (−14.78 to 7.24) | 0.837 |
| Diabetes | Social Functioning | 514 | −4.49 (−19.59 to 10.61) | 0.837 |
| Diabetes | Role-Emotional | 511 | 4.50 (−11.72 to 20.72) | 0.837 |
| Diabetes | Mental Health | 514 | −0.20 (−9.57 to 9.16) | 0.966 |
| Diabetes | Physical Component Summary | 508 | −3.63 (−9.45 to 2.18) | 0.734 |
| Diabetes | Mental Component Summary | 508 | 0.67 (−4.06 to 5.40) | 0.867 |
| Depressive disorder | Physical Functioning | 510 | −9.12 (−18.92 to 0.68) | 0.085 |
| Depressive disorder | Role-Physical | 507 | −16.41 (−29.60 to −3.23) | 0.025 |
| Depressive disorder | Bodily Pain | 509 | −9.05 (−20.00 to 1.90) | 0.117 |
| Depressive disorder | General Health | 510 | −13.23 (−20.40 to −6.05) | <0.001 |
| Depressive disorder | Vitality | 510 | −9.75 (−17.88 to −1.61) | 0.027 |
| Depressive disorder | Social Functioning | 510 | −17.54 (−26.95 to −8.12) | <0.001 |
| Depressive disorder | Role-Emotional | 507 | −28.44 (−40.91 to −15.98) | <0.001 |
| Depressive disorder | Mental Health | 510 | −19.06 (−25.22 to −12.89) | <0.001 |
| Depressive disorder | Physical Component Summary | 504 | −2.30 (−6.54 to 1.93) | 0.286 |
| Depressive disorder | Mental Component Summary | 504 | −10.08 (−13.30 to −6.87) | <0.001 |
| Non-skin cancer | Physical Functioning | 513 | 0.32 (−11.31 to 11.94) | 0.968 |
| Non-skin cancer | Role-Physical | 510 | 1.53 (−17.97 to 21.03) | 0.968 |
| Non-skin cancer | Bodily Pain | 512 | 7.00 (−3.70 to 17.71) | 0.968 |
| Non-skin cancer | General Health | 513 | 0.20 (−9.58 to 9.98) | 0.968 |
| Non-skin cancer | Vitality | 513 | 4.41 (−4.41 to 13.23) | 0.968 |
| Non-skin cancer | Social Functioning | 513 | 1.71 (−8.73 to 12.16) | 0.968 |
| Non-skin cancer | Role-Emotional | 510 | 3.47 (−8.83 to 15.77) | 0.968 |
| Non-skin cancer | Mental Health | 513 | 0.97 (−6.03 to 7.97) | 0.968 |
| Non-skin cancer | Physical Component Summary | 507 | 0.94 (−4.65 to 6.53) | 0.968 |
| Non-skin cancer | Mental Component Summary | 507 | 1.01 (−2.13 to 4.15) | 0.968 |
| Kidney disease | Physical Functioning | 514 | −2.73 (−20.72 to 15.26) | 0.915 |
| Kidney disease | Role-Physical | 511 | 12.50 (−11.41 to 36.42) | 0.915 |
| Kidney disease | Bodily Pain | 513 | 2.31 (−11.74 to 16.37) | 0.915 |
| Kidney disease | General Health | 514 | −2.53 (−13.05 to 8.00) | 0.915 |
| Kidney disease | Vitality | 514 | 4.64 (−2.50 to 11.78) | 0.915 |
| Kidney disease | Social Functioning | 514 | −6.19 (−21.54 to 9.17) | 0.915 |
| Kidney disease | Role-Emotional | 511 | −1.33 (−25.79 to 23.14) | 0.915 |
| Kidney disease | Mental Health | 514 | 4.78 (−2.01 to 11.57) | 0.915 |
| Kidney disease | Physical Component Summary | 508 | 0.44 (−6.64 to 7.53) | 0.915 |
| Kidney disease | Mental Component Summary | 508 | 0.57 (−6.00 to 7.14) | 0.915 |

***Note.*** *Models adjusted for age, sex, race/ethnicity, education, employment, rurality, and obesity. Obesity was defined as BMI ≥30 kg/m². Obesity itself is not listed as an exposure because a model cannot adjust an obesity exposure for obesity. Compare these estimates with Supplementary Table S1.*

# **Supplementary Table S5.** Separate associations of current asthma and COPD with SF-36 outcomes

| **Respiratory condition** | **SF-36 outcome** | **Analytic n** | **Adjusted β (95% CI)** | **qFDR** |
| --- | --- | --- | --- | --- |
| Current asthma (separate) | Physical Functioning | 535 | −16.28 (−29.55 to −3.01) | 0.027 |
| Current asthma (separate) | Role-Physical | 532 | −26.52 (−43.78 to −9.26) | 0.009 |
| Current asthma (separate) | Bodily Pain | 534 | −17.36 (−27.48 to −7.24) | 0.004 |
| Current asthma (separate) | General Health | 535 | −12.89 (−22.48 to −3.31) | 0.019 |
| Current asthma (separate) | Vitality | 535 | −10.20 (−18.93 to −1.47) | 0.032 |
| Current asthma (separate) | Social Functioning | 535 | −15.52 (−27.24 to −3.80) | 0.019 |
| Current asthma (separate) | Role-Emotional | 532 | −5.74 (−20.53 to 9.04) | 0.446 |
| Current asthma (separate) | Mental Health | 535 | −5.40 (−14.24 to 3.44) | 0.288 |
| Current asthma (separate) | Physical Component Summary | 529 | −8.84 (−13.60 to −4.08) | 0.003 |
| Current asthma (separate) | Mental Component Summary | 529 | −2.03 (−6.67 to 2.62) | 0.436 |
| COPD (separate) | Physical Functioning | 534 | −8.12 (−24.16 to 7.92) | 0.534 |
| COPD (separate) | Role-Physical | 531 | −11.98 (−33.46 to 9.51) | 0.534 |
| COPD (separate) | Bodily Pain | 534 | −6.08 (−24.67 to 12.52) | 0.667 |
| COPD (separate) | General Health | 534 | −12.21 (−21.65 to −2.78) | 0.056 |
| COPD (separate) | Vitality | 534 | −3.03 (−14.37 to 8.31) | 0.667 |
| COPD (separate) | Social Functioning | 534 | −5.23 (−21.81 to 11.35) | 0.667 |
| COPD (separate) | Role-Emotional | 532 | −0.77 (−18.84 to 17.30) | 0.933 |
| COPD (separate) | Mental Health | 534 | −13.65 (−23.52 to −3.77) | 0.056 |
| COPD (separate) | Physical Component Summary | 529 | −3.36 (−9.65 to 2.93) | 0.534 |
| COPD (separate) | Mental Component Summary | 529 | −3.16 (−7.97 to 1.65) | 0.534 |

***Note.*** *Models adjusted for age, sex, race/ethnicity, education, employment, and rurality. Confirm the CATI codebook labels for the asthma and COPD items before submission.*

# **Supplementary Table S6A.** Exploratory weighted estimates by rurality

| **Rurality** | **Unweighted n** | **Multimorbidity, weighted % (95% CI)** | **PCS mean (95% CI)** | **MCS mean (95% CI)** |
| --- | --- | --- | --- | --- |
| Urban or metro | 328 | 28.7 (19.8 to 37.7) | 46.5 (44.0 to 48.9) | 50.2 (48.3 to 52.2) |
| Rural or nonmetro | 191 | 36.3 (23.6 to 49.1) | 44.2 (40.7 to 47.7) | 50.6 (48.4 to 52.8) |
| Unknown | 28 | 21.1 (3.0 to 39.2) | 51.3 (47.8 to 54.8) | 48.0 (41.6 to 54.3) |

***Note.*** *Multimorbidity was defined as two or more of the seven primary conditions. Estimates are descriptive and survey weighted.*

# **Supplementary Table S6B.** Exploratory weighted estimates by race/ethnicity

| **Race/ethnicity** | **Unweighted n** | **Multimorbidity, weighted % (95% CI)** | **PCS mean (95% CI)** | **MCS mean (95% CI)** |
| --- | --- | --- | --- | --- |
| Non-Hispanic White | 445 | 33.8 (25.5 to 42.2) | 45.3 (42.9 to 47.6) | 50.3 (48.7 to 51.9) |
| Non-Hispanic Black | 49 | 13.2 (1.9 to 24.6) | 50.5 (46.8 to 54.3) | 47.6 (42.8 to 52.5) |
| Hispanic | 15 | 36.3 (-2.8 to 75.3) | 50.9 (46.7 to 55.0) | 51.2 (43.6 to 58.7) |
| Non-Hispanic Other | 36 | 6.2 (-1.6 to 14.1) | 48.3 (42.5 to 54.0) | 53.4 (45.4 to 61.5) |
| Unknown | 2 | 73.9 (20.4 to 127.4) | 29.4 (19.5 to 39.3) | 38.1 (33.7 to 42.5) |

***Note.*** *Estimates for small groups, particularly the unknown category, are unstable and should not be emphasized.*

# **Supplementary Table S6C.** Exploratory interaction tests for chronic disease burden by rurality and race/ethnicity

| **Outcome** | **Modifier** | **Test df** | **Analytic n** | **Interaction p** |
| --- | --- | --- | --- | --- |
| Physical Component Summary | Rurality (3 categories) | 4 | 535 | 0.119 |
| Mental Component Summary | Rurality (3 categories) | 4 | 535 | 0.379 |
| Physical Component Summary | Race/ethnicity (White vs minority) | 2 | 535 | 0.514 |
| Mental Component Summary | Race/ethnicity (White vs minority) | 2 | 535 | 0.968 |
| Physical Component Summary | Race/ethnicity (4 categories) | 6 | 535 | 0.002 |
| Mental Component Summary | Race/ethnicity (4 categories) | 6 | 535 | 0.113 |

***Note.*** *Interaction tests were design-based Wald tests. The four-category race/ethnicity interaction corresponds to the primary race/ethnicity variable; the White-versus-minority result is an additional collapsed exploratory specification.*

# **Supplementary Table S7.** Exploratory age-stratified associations of chronic disease burden with Physical Functioning, PCS, and MCS

| **Age group** | **Outcome** | **Contrast** | **Analytic n** | **Adjusted β (95% CI)** | **Raw p** |
| --- | --- | --- | --- | --- | --- |
| 18-44 | Physical Functioning | 1 vs 0 conditions | 182 | −2.89 (−7.88 to 2.09) | 0.253 |
| 18-44 | Physical Functioning | ≥2 vs 0 conditions | 182 | −23.86 (−38.64 to −9.09) | 0.002 |
| 18-44 | Physical Component Summary | 1 vs 0 conditions | 181 | −2.90 (−5.34 to −0.46) | 0.020 |
| 18-44 | Physical Component Summary | ≥2 vs 0 conditions | 181 | −11.82 (−17.63 to −6.00) | <0.001 |
| 18-44 | Mental Component Summary | 1 vs 0 conditions | 181 | −3.02 (−7.69 to 1.64) | 0.202 |
| 18-44 | Mental Component Summary | ≥2 vs 0 conditions | 181 | −6.97 (−13.23 to −0.71) | 0.029 |
| 45-64 | Physical Functioning | 1 vs 0 conditions | 150 | −12.68 (−32.04 to 6.68) | 0.197 |
| 45-64 | Physical Functioning | ≥2 vs 0 conditions | 150 | −29.32 (−45.08 to −13.56) | <0.001 |
| 45-64 | Physical Component Summary | 1 vs 0 conditions | 149 | −5.98 (−13.70 to 1.74) | 0.128 |
| 45-64 | Physical Component Summary | ≥2 vs 0 conditions | 149 | −12.87 (−17.94 to −7.81) | <0.001 |
| 45-64 | Mental Component Summary | 1 vs 0 conditions | 149 | 2.82 (−1.76 to 7.40) | 0.225 |
| 45-64 | Mental Component Summary | ≥2 vs 0 conditions | 149 | −7.40 (−11.51 to −3.30) | <0.001 |
| 65+ | Physical Functioning | 1 vs 0 conditions | 209 | −13.20 (−25.57 to −0.83) | 0.037 |
| 65+ | Physical Functioning | ≥2 vs 0 conditions | 209 | −33.25 (−46.97 to −19.53) | <0.001 |
| 65+ | Physical Component Summary | 1 vs 0 conditions | 205 | −4.84 (−10.78 to 1.11) | 0.110 |
| 65+ | Physical Component Summary | ≥2 vs 0 conditions | 205 | −13.12 (−19.29 to −6.95) | <0.001 |
| 65+ | Mental Component Summary | 1 vs 0 conditions | 205 | −2.62 (−6.47 to 1.23) | 0.180 |
| 65+ | Mental Component Summary | ≥2 vs 0 conditions | 205 | −6.18 (−9.55 to −2.81) | <0.001 |

***Note.*** *Models were fitted separately within each age group and adjusted for continuous age within the stratum, sex, race/ethnicity, education, employment, and rurality. These estimates are exploratory and were not FDR corrected. Some exposure cells were small.*

# **Supplementary Table S8.** Scale-specific variability and standardized adjusted differences

| **SF-36 outcome** | **Full-sample SD** | **Half SD** | **Zero-condition SD** | **Half SD in zero-condition group** | **Adjusted β, ≥2 vs 0** | **β / zero-condition SD** |
| --- | --- | --- | --- | --- | --- | --- |
| Physical Functioning | 28.19 | 14.10 | 15.31 | 7.66 | −28.70 | −1.87 |
| Role-Physical | 39.85 | 19.93 | 30.19 | 15.10 | −30.65 | −1.02 |
| Bodily Pain | 27.09 | 13.55 | 18.40 | 9.20 | −28.28 | −1.54 |
| General Health | 21.29 | 10.64 | 15.36 | 7.68 | −24.18 | −1.57 |
| Vitality | 23.18 | 11.59 | 21.34 | 10.67 | −20.56 | −0.96 |
| Social Functioning | 26.19 | 13.09 | 17.16 | 8.58 | −26.18 | −1.53 |
| Role-Emotional | 33.04 | 16.52 | 30.08 | 15.04 | −11.03 | −0.37 |
| Mental Health | 18.11 | 9.06 | 15.07 | 7.54 | −16.29 | −1.08 |
| Physical Component Summary | 12.34 | 6.17 | 7.46 | 3.73 | −12.82 | −1.72 |
| Mental Component Summary | 10.09 | 5.05 | 8.87 | 4.43 | −6.10 | −0.69 |

***Note.*** *Standardized differences were calculated by dividing the adjusted ≥2-versus-0 coefficient by the survey-weighted SD among adults with no chronic conditions. These values describe cross-sectional between-group differences and should not be interpreted as minimally important within-person changes.*

# **Supplementary Table S9.** Disposition counts and AAPOR Response Rate 4 calculation

| **Disposition category** | **BRFSS codes** | **Landline** | **Cell phone** | **Total** |
| --- | --- | --- | --- | --- |
| **Total telephone numbers released** | All records | **4,009** | **12,832** | **16,841** |
| Complete interviews | 1100 | 131 | 416 | **547** |
| Partial interviews | 1200 | 0 | 0 | **0** |
| Refusals and break offs | 2111, 2112, 2120 | 131 | 247 | **378** |
| Noncontact, selected respondent never available | 2210 | 15 | 85 | **100** |
| Other eligible noninterview | 2320, 2330 | 2 | 4 | **6** |
| **Total eligible, ELIG** | 1100, 1200, 2111, 2112, 2120, 2210, 2320, 2330 | **279** | **752** | **1,031** |
| **Unknown eligibility, UNKELIG** | All 3000 level codes | **1,854** | **8,018** | **9,872** |
| **Not eligible, INELIG** | All 4000 level codes | **1,876** | **4,062** | **5,938** |
| Eligibility factor, e | ELIG/(ELIG + INELIG) | **0.1295** | **0.1562** | NAᵃ |
| **AAPOR RR4, %** | COIN/[ELIG + (e × UNKELIG)] × 100 | **25.2** | **20.8** | **21.8ᵇ** |

ᵃ The eligibility factor (e) was calculated separately for the landline and cellular telephone frames as the proportion eligible among telephone numbers for which eligibility was determined.
ᵇ The overall response rate was calculated as the sample size weighted combination of the landline and cellular telephone response rates, consistent with CDC BRFSS methodology.

COIN represents completed plus partial interviews included in the RR4 numerator. In this survey, there were no partial interviews; therefore, COIN equaled the number of completed interviews.

**Supplementary Table S10.** Reporting of complex sample survey analysis using PRICSSA items

| **PRICSSA item** | **How this study addresses the item** | **Manuscript location** |
| --- | --- | --- |
| 1.1 Data collection dates | The manuscript identifies the 2024 CMEPH Adult Health Survey | Methods, Study Design and Population |
| 1.2 Data collection mode(s) | Trained interviewers administered the survey by computer-assisted telephone interviewing (CATI). Separate landline and cellular telephone random digit dialing frames were used, and responses were self reported by sampled adults. | Methods, Study Design and Population |
| 1.3 Target population | The target population is noninstitutionalized Missouri adults aged 18 years or older. Weighted estimates are interpreted for this population. The final weighted population represented 4,809,135 Missouri adults. | Methods, Study Design and Population; Discussion, Limitations |
| 1.4 Sample design | The survey used a dual frame random digit dialing design with separate landline and cellular telephone frames. The design incorporated geographic and telephone number density strata. The analytic survey design used STRATUM and the final survey weight. | Methods, Study Design and Population |
| 1.5 Survey response rate(s) | AAPOR Response Rate 4 was 21.8% overall, 25.2% for landline, and 20.8% for cellular telephone. Supplementary Table S9 provides frame-specific disposition counts, eligibility factors, and the RR4 calculation so the estimates can be reproduced. | Methods, Study Design and Population; Supplementary Table S9 |
| 2.1 Missingness rates | Missingness is reported through variable-specific denominators, analytic sample sizes, and the supplementary missingness audit. Six respondents had sparse unknown values for race/ethnicity, education, or employment; 28 had unknown rurality. Outcome-specific missingness is reflected in analytic n. No additional imputation was performed by the analytic team; survey-provided imputed demographic variables were used for sex and race where applicable. | Table 1; Table 2; Table 3; Methods, Statistical Analysis; supplementary tables |
| 2.2 Observation deletion | Six respondents with sparse unknown race/ethnicity, education, or employment values were excluded from primary regression models because these categories were too small for stable adjustment. Unknown rurality was retained in the primary analysis, with a complete case sensitivity analysis that additionally excluded 28 respondents with unknown rurality. | Methods, Statistical Analysis; Supplementary Table S2B |
| 2.3 Sample sizes | Unweighted sample sizes are reported for the main weighted descriptive estimates and primary models. Table 2 reports n for every burden group and Table 3 now reports outcome-specific analytic n. Most supplementary model tables also report analytic n. | Tables 1 to 3; Supplementary Tables S1 to S8 |
| 2.4 Confidence intervals / standard errors | Regression estimates and most supplementary weighted estimates include 95% confidence intervals or standard errors. Table 2 now reports survey design based 95% confidence intervals in addition to weighted SDs and unweighted n. | Tables 1 to 3; supplementary tables |
| 2.5 Weighting | Population estimates and regression analyses use the final analytic survey weight _LLCPWT. Unweighted counts and percentages are reported descriptively to show the observed sample composition. Weighted analyses also incorporate STRATUM. | Methods, Study Design and Population; Methods, Statistical Analysis |
| 2.6 Variance estimation | Variance estimation uses Taylor series linearization through the R survey package. The analytic survey design is specified with STRATUM and _LLCPWT, with respondents treated as sampling units because no separate PSU variable is used in the analytic design object. | Methods, Statistical Analysis |
| 2.7 Subpopulation analysis | Age subgroup models use survey-aware subsetting of the design object so the original survey design information is retained. Rurality and race/ethnicity descriptive subgroup estimates use survey domain estimation, and effect modification is evaluated with survey-weighted interaction models and design based Wald tests. | Methods, Statistical Analysis; Supplementary Tables S6A to S6C and S7 |
| 2.8 Suppression rules | No formal suppression rule was applied. Unweighted subgroup sizes are reported, estimates from sparse cells are labeled exploratory, and small subgroup results are interpreted cautiously rather than presented as stable population estimates. | Methods, Statistical Analysis; Results, Exploratory analyses |
| 2.9 Software and code | The R code used for data management and statistical analysis is available from the corresponding author upon reasonable request. | Methods, Statistical Analysis; Data and Code Availability statement |
| 2.10 Singleton problem (as needed) | Some strata contained a single sampling unit. The survey package lonely PSU setting was set to "adjust", which centers singleton strata using the grand mean for variance estimation. | Methods, Statistical Analysis |
| 2.11 Public / restricted data (as needed) | The CMEPH Adult Health Survey analytic data are not publicly available. Data used for this study may be made available from the corresponding author upon request, subject to applicable institutional requirements. | Data Availability statement |
| 2.12 Embedded experiments (as needed) | No split sample embedded experiment is described in the survey or analysis. Landline versus cellular telephone is a sampling frame feature addressed through the survey design and weighting, not an experimental assignment. | Not applicable |

Reference: Seidenberg AB, Moser RP, West BT. Preferred Reporting Items for Complex Sample Survey Analysis (PRICSSA). Journal of Survey Statistics and Methodology. 2023;11(4):743-757. doi:10.1093/jssam/smac040.
