# Supplementary material for: Chronic disease burden and health-related quality of life among missouri adults
Source: Qual Life Res. 2026 Nov 1;35(11):318. doi: 10.1007/s11136-026-04422-6 (PMC13724639; doi:10.1007/s11136-026-04422-6)
Supplement: Supplementary file 2 [file 11136_2026_4422_MOESM2_ESM.docx]

**Supplementary Figure S1.** Adjusted ≥2-versus-0 differences in SF-36 outcomes using linear and flexible age specifications
